# Microplastic stress in plants: effects on plant growth and their remediations

**DOI:** 10.3389/fpls.2023.1226484

**Published:** 2023-08-11

**Authors:** Li Jia, Lining Liu, Yujing Zhang, Wenxuan Fu, Xing Liu, Qianqian Wang, Mohsin Tanveer, Liping Huang

**Affiliations:** ^1^ College of Food and Drug, Luoyang Normal University, Luoyang, Henan, China; ^2^ International Research Center for Environmental Membrane Biology, College of Food Science and Engineering, Foshan University, Foshan, China; ^3^ Tasmanian Institute of Agriculture, University of Tasmania, Hobart, TAS, Australia

**Keywords:** microplastics, root and shoot physiology, photosynthesis, ROS, ionic homeostasis, gene expression, phytohormonal regulation

## Abstract

Microplastic (MP) pollution is becoming a global problem due to the resilience, long-term persistence, and robustness of MPs in different ecosystems. In terrestrial ecosystems, plants are exposed to MP stress, thereby affecting overall plant growth and development. This review article has critically analyzed the effects of MP stress in plants. We found that MP stress-induced reduction in plant physical growth is accompanied by two complementary effects: (i) blockage of pores in seed coat or roots to alter water and nutrient uptake, and (ii) induction of drought due to increased soil cracking effects of MPs. Nonetheless, the reduction in physiological growth under MP stress is accompanied by four complementary effects: (i) excessive production of ROS, (ii) alteration in leaf and root ionome, (iii) impaired hormonal regulation, and (iv) decline in chlorophyll and photosynthesis. Considering that, we suggested that targeting the redox regulatory mechanisms could be beneficial in improving tolerance to MPs in plants; however, antioxidant activities are highly dependent on plant species, plant tissue, MP type, and MP dose. MP stress also indirectly reduces plant growth by altering soil productivity. However, MP-induced negative effects vary due to the presence of different surface functional groups and particle sizes. In the end, we suggested the utilization of agronomic approaches, including the application of growth regulators, biochar, and replacing plastic mulch with crop residues, crop diversification, and biological degradation, to ameliorate the effects of MP stress in plants. The efficiency of these methods is also MP-type-specific and dose-dependent.

## 1 Introduction

**Figure 1:**
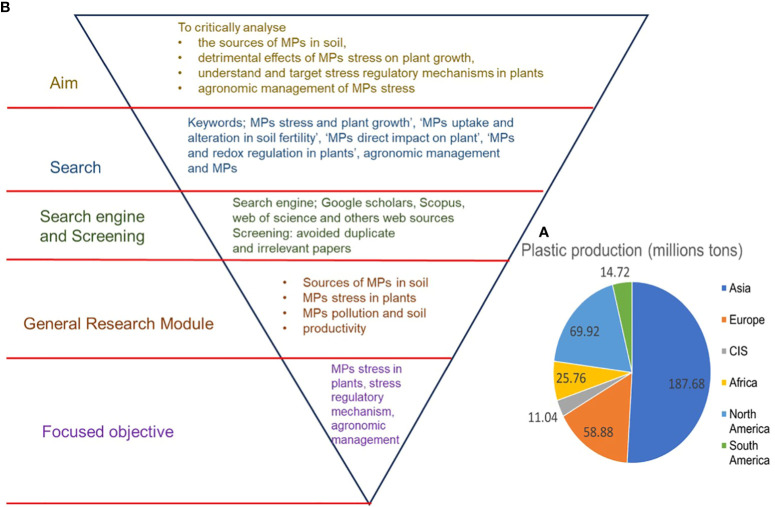
**(A)** worldwide plastic production (Plastic Europe 2020) and **(B)** flowchart of the journal article decision-making process and scope of the review.

The post-industrialization era brought drastic global plastic production, which was essential at that time, however, since the last two decades, excess plastic production and improper disposal methods have led to plastic pollution. Global plastic production reached 368 million tons in 2019 and is expected to double in the next 20 years (Huang et al., 2021). Out of the total of 368 million tons of plastic produced globally, Asia is the biggest producer, contributing 187.68 million tons of plastic, followed by Europe with 58.88 million tons (**Figure 1A**) (Plastics Europe, 2020). However, approximately only 26% of total plastic produced is recycled, with the rest ending up either in landfills or entering the environment via various means (Azeem et al., 2021; Huang et al., 2021). In different environmental settings, including terrestrial environments, plastics undergo fragmentation and decompose into particles with different sizes: microplastics (MPs) (>25 mm), mesoplastics (5–25 mm), and nanoplastics (NPs) (<100 nm) (Alimi et al., 2018; Azeem et al., 2021). MPs persist in the environment and can remain there for hundreds of years (Duan et al., 2021). MPs have been found in various shapes, sizes, polymers, and concentrations in agroecosystems, in terrestrial and aquatic settings (Azeem et al., 2022; Pan et al., 2021; Mao et al., 2022). Moreover, MPs have been observed in different food items that are widely consumed by humans, such as table salt, seafood, vegetables, and grains (Campanale et al., 2022; Kibria et al., 2022; Pan et al., 2022; Udovicki et al., 2022; Makhdoumi et al., 2023). Given the increasing accumulation of MPs in soil, which has reached a level that cannot be ignored (Makhdoumi et al., 2023), it is important to study MP-induced toxic effects on plants since the higher uptake of MPs by edible plants may pose serious health risks to humans (Sridharan et al., 2020; Pan et al., 2022).

Generally, once MPs enter the soil, they persist, accumulate, and then gradually disturb the functioning and biodiversity of soil ecosystems (Guo et al., 2019; Kumar et al., 2020). For instance, soil physicochemical properties have changed with the increasing concentrations of MPs, which led to a change in enzyme activities and microbial biodiversity (De Souza Machado et al., 2019; Kumar et al., 2020). MPs also interact with the soil environment and alter the soil bulk density by affecting the stability of soil aggregates, which are building blocks of the soil structure (Rillig et al., 2021). Such MP-induced alteration on soil fertility imposes indirect negative effects on plant growth. MPs affect soil fertility with subsequent alteration in plant growth by altering root growth and nutrient uptake (Ge et al., 2021; Zhou et al., 2023).

MP stress also reduces plant growth and development by altering several key physiological processes including ionic homeostasis, redox regulation, and photosynthesis (Guo et al., 2022; Maity et al., 2022). Nonetheless, MP-induced negative effects on plant growth and development depend on MP type- and dose-dependent effects (Jiang et al., 2019; Wang et al., 2020a; Ge et al., 2021). In plants, MP stress has a direct interaction with the plant roots because MPs can be adsorbed onto root hairs, thus affecting root growth (Bosker et al., 2019). Whereas in leaves, MP stress most likely causes oxidative stress, thus reducing leaf growth and photosynthesis (Lian et al., 2021; Colzi et al., 2022). Nonetheless, there is no explicit explanation of how MPs induce toxic effects on plant growth and development.

This review has compiled and reviewed all the published literature relating to MP stress in plants. The direct and indirect impacts of MPs on plant growth and development have also been analyzed. Moreover, targeting several key stress regulatory physiological mechanisms is suggested to improve plants’ tolerance to MPs or at least lead to better performance under MP stress. Thus, this review aims to critically analyze the direct and indirect impacts of MPs on plant growth and development. In addition, the agronomic management of MP stress including the application of plant growth regulators, biochar, and biodegradation in plants has also been discussed for the sustainable remediation of MP stress in plants.

## 2 Literature search

This review article analyzed the literature through the databases of Google Scholars, Web of Science, and other web sources using keywords such as ‘MP toxicity and plant growth’, ‘MPs uptake and seed germination’, ‘MPs and alteration in soil fertility’, ‘MPs direct impact on plant’, ‘MPs and root cell wall’, ‘MPs and redox regulation in plants’, MPs uptake and translocation in plants, ‘MPs and biochar application and plant growth’, ‘plant growth regulators and MPs toxicity in plants’, and ‘Biodegradation of MPs’ (**Figure 1B**). This review outlines the areas that require further attention to fill the current knowledge gap. Moreover, increased knowledge about MP toxicity in plants and their amelioration using agronomic means will benefit researchers and farmers by changing the public perception of MP toxicity in plants and by the development of MP stress-tolerant traits in plants.

## 3 Sources and distribution of microplastics in soil

Plastic use has increased rapidly since synthetic organic polymers were developed in the mid-20^th^ century. In recent decades, the annual global production of plastics has increased rapidly and reached approximately 359 M tons (Plastics Europe, 2019). Moreover, if plastic production were to remain uncontrolled, its production would reach 670 MT by 2040 (Delangiz et al., 2022). However, it may not be appropriate to assume that more production of plastic necessarily means more MPs in the environment, given the significance of ongoing developments and improvements in recycling processes and the replacement of plastics in different industrial compartments (Law and Narayan, 2022). Nevertheless, the global release of plastics due to their mismanagement has reached almost 4.8-12.7 M tons every year (Jambeck et al., 2015). In the terrestrial ecosystem, approximately 79% of the waste in landfills is plastic (Geyer et al., 2017), and soil is a major sink for MP pollution in the terrestrial ecosystem. MPs enter the soil environment via various pathways (**Table 1**), including the application of sewage sludge or soil amendments with compost, irrigation with MP-contaminated water, plastic mulching, atmospheric deposition, and littering (Bläsing and Amelung, 2018; Kumar et al., 2022). Recently, Hernández-Arenas et al. (2021) found up to 31,000 ± 8600 particles kg^−1^ dry weight of MPs in sewage sludge, and most of the particles were PET, PP, LDPE, and HDPE. MPs transfer in soil via the uptake by either plant roots or by soil microorganisms (e.g., earthworms, vertebrates). Earthworms play a significant role in the transport of MPs from topsoil to deep soils as they can absorb pollutants from the substrate through ingestion or body wall (Adeel et al., 2019; Adeel et al., 2021). For instance, anecic earthworms make permanent vertical burrows in soil with subsequent translocation of MPs from the topsoil to deep soils (Hurley and Nizzetto, 2018). Though the use of plastic mulch has a significant role in retaining soil water under water deficit conditions, the poor management of plastic mulch results in the addition of plastics in the soil (Huang et al., 2020). Globally, plastic mulch use has increased from 4.4 million tons in 2012 to 7.4 million tons in 2019 (Li et al., 2019). The use of PAEs in plastic mulch enhances the risk to many agricultural products (Viljoen et al., 2023). Biodegradable plastic films are unlikely to create more problems in terms of environmental damage (Sintim et al., 2019).

**Table 1:** Attributes of MPs derived from different sources in soil.

Sources	Type	Size	Abundance	References
***Sewage waste***	PP, PVC	>1 mm, 50-500μm	2130 ± 950 items per kg	van den Berg et al., 2020
	PVC	Up to 500μm	3060 ± 1680 items per kg	van den Berg et al., 2020
	PE	0.05 - 1 mm	18,760 particles per kg	Zhang et al., 2018
	Polyolefin, acrylic fibers	37 μm - 5 mm	22.7 ± 12.1 ×10^3^ particles per kg	Li et al., 2018
***Compost***	PS, PE, PES, PET, PP, PVC, and PA	1 - 5 mm	11 - 895 particles per kg	Weithmann et al., 2018
	PE, PP, and PES	< 2 mm- 5 mm	3.50 ± 1.71 × 10^6^ particles per hectare	Yang et al., 2021a
***Mulching films***	PA,	< 2mm	320-12,560 particles per kg	Chen et al., 2020
	PP	20 μm	62.50 particles per kg	Liu et al., 2018
	PP	< 2mm	320-12,560 particles per kg	Chen et al., 2020
	PE	5mm	78.00 particles per kg	Liu et al., 2018
	PE	7 μm - 5000 μm	1075.6 particles per kg	Huang et al., 2020
	PE	100 - 800 μm	9.25 - 369.55 mg per kg	Li et al., 2020

Sludge is used as fertilizer on agricultural fields, which also creates another primary driver of soil MP pollution. Up to 2005, there was evidence demonstrating that synthetic fibers accumulate in soils treated with sludge (Corradini et al., 2019). Likewise, a high concentration of MPs was detected in compost, which implies that there is another important pathway for MPs in agricultural soils. The average accumulation of MPs in agricultural soils during long-term repeated application of compost could be up to 3.30 million particles/ha/year (Yang et al., 2021a), which is due to the high temperature and microbial activities during the composting process. Moreover, the brittleness of plastics often increases after fermentation (Accinelli et al., 2022), and the mechanical behaviors of fertilizer production, including crushing, granulation, drying, cooling, and sieving, may contribute to the further fragmentation of macroplastics and the formation of microplastics (Braun et al., 2021). MPs migrate from topsoil to subsoil in response to tillage and harvesting (Huang et al., 2021). Moreover, the infiltration process of water flow such as rainfall or irrigation can stimulate the migration of MP transfer in soil voids (Wong et al., 2020). Soil acts as one of the major sinks for MP accumulation in terrestrial ecosystems; however, growing evidence shows a large variation in the abundance of MPs and their source of transport in different soils such as agricultural soils, marginal soils, industrial soils, and urban soils (Zhang and Liu, 2018). These studies showed that the abundance of MPs depends on the composition, size, shape, and source of MP pollution in soil, thereby data on MP abundance in soil cannot be directly compared. Such lack of information also influences the understanding of MP stress in plants.

## 4 Microplastic effects on plant growth

Plant growth can be defined as a process of increasing plant volume or mass with or without the development of new structures (e.g., organs, cells, or tissues). This process is associated with physical cell specialization and reproduction and physiological processes; however, this process is highly sensitive to growth conditions. Any alteration in growth conditions from optimal growth conditions results in the alteration of these mechanisms, thus thereby reducing plant growth and development. MP stress reduces plant growth and development by imposing (i) direct effects linked with the physical obstruction, and (ii) indirect effects associated with a reduction in soil productivity. In this section, we have discussed the direct and indirect effects of MP stress on plant growth and development and highlighted the knowledge gaps that need to be filled in future studies. Moreover, the uptake and translocation of MPs from root to shoot/leaves have also been discussed accordingly in this section.

### 4.1 Direct effects

#### 4.1.1 Physical growth reduction

***Seed germination*** is the first phase in plant growth and is highly sensitive to stress conditions (Wang and Tanveer, 2023) including MP stress (**Table 2**). MP stress reduces seed germination by clogging the pores in the seed capsule (Bosker et al., 2019), thus reducing water uptake and the imbibition process. Reduction in the imbibition process results in a reduced germination rate (Zhang et al., 2021). The seed germination rates and germination potential of three herbaceous ornamental plants, *Trifolium repens*, *Orychophragmus violaceus*, and *Impatiens balsamina*, were reduced in response to PS MP stress (Guo et al., 2022). Co-exposure of polymethyl methacrylate (PMMA) MPs and As (V) reduced the germination index, root length, and sprout length of rapeseed (*Brassica campestris* L.) (Dong et al., 2022a). Nonetheless, De Silva et al. (2022) argued that examining the conventional parameters of seed germination, including germination rate and germination viability, could not be reliable to examine the direct effects of MP stress on seed germination. As such measurements require destructive post-harvesting measurements, the use of techniques such as biospeckle optical coherence tomography (BOCT) can provide an explicit picture of the effects of MP stress on seed germination. In this context, De Silva et al. (2022) used BOCT and observed that PE MPinduced reduction in seed germination was associated with the reduction in the internal biological activity, most likely due to physical blockage of pores in seeds (Zhang et al., 2021), MP-induced cytotoxicity during SG (Bao et al., 2022), or due to the presence of plasticizers in MPs (Balestri et al., 2019). However, such alterations could be MPs dose-, plant species- and exposure time-dependent (Sahasa et al., 2023).

**Table 2:** Selected examples (published in the last three years) of the reported effects of different microplastics (MPs) on plant growth and development.

MP type and concentration	Plant Species	Effects on plant growth and development	Reference
PE (0.0125 and100 mg·L^-1^)	Maize	• Impaired nitrogen uptake • Impaired water uptake • Reduced overall growth	Urbina et al., 2020
PS (0.5-3 mg L^-1^) and PVC (0.5-3 mg L^-1^)	Rice	• Reduced photosynthesis, leaf gas exchange • Reduced chlorophyll contents • Impaired nutrient uptake	Ma et al., 2022
PS (0.1-10 mg L^−1^)	Rice	• Triggered phytotoxicity • Enhanced oxidative damage	Wu et al., 2021
PC (0.1-10% (w/w)	Garden cress	• Reduced seed germination • Decreased root and shoot length	Pflugmacher et al., 2020
HDPE (3% w/w)	Rye grass (*Lolium multiflorum)*	• Regardless of the ages of HDPE particles, HDPE particles reduced seed germination and root-shoot length.	Esterhuizen et al., 2022
PET and PEF (0.5-2%	Lettuce	• Impaired lettuce growth • Enhanced ROS production • Reduced soluble sugar and nitrogen accumulation	Zhang et al., 2022
PS (0.01-10 mg/L	Wheat	• Reduced the shoot-to-root biomass ratio • Impaired micronutrients contents	Lian et al., 2020
PS (25 - 400 mg/L)	Onion	• Reduced root length • Caused cytogenetic toxicity • Enhanced ROS production • Inhibited the expression of *cdc*2	Maity et al., 2020
PP, HDPE, PS, PVC, PET, PUR (20%)	Lotus (*Nelumbo nucifera)*	• The germination rate is reduced	Esterhuizen and Kim, 2022
PS (1, 10 mgL^-1^)	Italian lettuce, radish, wheat, and corn	• Reduced root dry weight • Reduced the root-to-shoot ratio	Gong et al., 2021
PS (0, 10, 50, 100, 500 mg/L)	White clover, Chinese violet cress, and Balsama	• Reduced germination potential and germination rate	Guo et al., 2022
PE (0.001%, 0.01%, 0.1% (w/w)	*Brassica napus* L.	• Reduced the total chlorophyll content, • Altered sugar metabolism, • Enhanced bioaccumulation of lead	Jia et al., 2022
Shoe sole fragments 0%, 0.1%, 1% (w/w)	*Mung bean*	• MP fragments and leachates from the soles of shoes affected plant growth	Lee et al., 2022
PE (0.01-10,000 mg L^-1^)	*Brassica oleracea*	• Induced oxidative damage	López et al., 2022
PE (0.1% (w/w)	*Maize*	• Reduced maize growth • Altered soil bacterial community • Affected gene expression of antioxidants	Fajardo et al., 2022
PMFs (0.1%, 0.2% (w/w))	*Lettuce*	• Reduced shoot length • Altered photosynthesis and chlorophyll contents • Altered carbohydrate and nitrogen metabolic pathway	Zeb et al., 2022
PS *Hydroponics*: (50-500 mg L^-1^) *Soil culture*: (50-500 mg L^-1^)	*Rice*	• Reduced shoot biomass and altered antioxidants activities under hydroponics • Decreased rice biomass production under soil culture	Wu et al., 2020
PS (0.3, 1.0 g/kg)	*Arabidopsis*	• Reduced fresh weight • Modify gene expression	Sun et al., 2020
PS (1 mg/kg)	*Soybean*	• Induced oxidative stress • Altered gene expression	Xu et al., 2021
PVC 100 and 200 mg/L	*Sweet potato*	• Decreased plant height, fresh biomass per plant Reduced chlorophyll content • PVC increased chromium accumulation in sweet potato	Khan et al., 2023
PVC 100 and 1000 mg/L	*Spirodela polyrhiza*	• Significant reduction in leaf multiplication • Declined adventitious root elongation, thereby reducing overall growth	Wang et al., 2023b

The degree of MP stress-induced reduction in seed germination also depends on the particle size of MPs. For instance, PS MPs with a particle size of 100 nm reduced tomato (*Lycopersicon esculentum* L.) seed germination in a relatively higher percentage when compared with PS MPs with a particle size of 5 μm (Liao et al., 2019), thereby indicating that MPs with large particle sizes reduce seed germination more significantly as compared with MPs with smaller particle sizes (Bosker et al., 2019; Wang et al., 2023a). Moreover, leachates produced during plastic degradation negatively affect seed germination. For instance, leachates of oxo-degradable PP reduced the germination of sorghum (*Sorghum bicolor* L.) (Schiavo et al., 2020). Likewise, leachates of PC showed negative effects on the germination of garden cress (Pflugmacher et al., 2020). However, MP-induced stimulatory effects on seed germination have also been reported (Lian et al., 2019). High concentrations of MPs in soil promoted wheat (*Triticum aestivum* L.) seed germination, while low and medium concentrations showed inhibitory effects on seed germination (Lian et al., 2019). This could be because of different particle sizes, agglomeration, and charge on MP particles, which may influence the ability of the wheat seed to germinate and grow under MP stress (Weber et al., 2018; Ziajahromi et al., 2018; Ge et al., 2021). Moreover, the seed germination of chickpea (*Cicer arietinum* L.) was increased after the exposure to PET MPs (Mondal et al., 2022), potentially due to the priming effects of MPs on seed germination or due to the ability of MPs to break seed coat and enhance the imbibition process due to better water uptake via microscopic pores.

***Roots*** are ranked second amongst other plant parts that face direct and mechanical damage under MP stress. MPs can accumulate in roots and reduce root elongation, root activity, and root fresh and dry biomass production (Boots et al., 2019; Maity et al., 2022; Wang et al., 2023a). MP stress-induced root growth reduction has been reported in several crops (**Table 2**), including wheat by LDPE MPs (Qi et al., 2018), broad bean (*Vicia faba* L.) and onion (*Allium cepa* L.) by PS MPs (Jiang et al., 2019; Lian et al., 2022), and broad bean by Bio-MP stress (Meng et al., 2022). Moreover, PS MPs can adhere to the root surface and cause physical blockage to root pores (Gao et al., 2021). Blockage of root pores by sharp-edged MPs can reduce root growth by altering water and nutrient uptake from soil. Moreover, exposure of roots to MPs resulted in physical damage to the root and induced oxidative stress (Dong et al., 2020), reduced root-branch ratio and root biomass (Qi et al., 2018), reduced nitrogen contents and transpiration rate (Urbina et al., 2020), and reduced respiration (Liu et al., 2019; Spano et al., 2022). A previous study found that PS MPs can accumulate in rice (*Oryza sativa* L.) root via the endocytosis mechanism. During the fragmentation of MPs, particle size gets smaller but gains more specific surface area, thus having more potential to be adsorbed on the root surface. However, MPs can also accumulate in the root via the endocytosis mechanism (Wu et al., 2021).

Some MPs, such as PS MPs, are hydrophobic and can easily be absorbed on the root surface, thus reducing root growth (Nel et al., 2009). Moreover, MP stress reduces nutrient uptake due to the hetero aggregation of opposite charges and pore blockage in the cell wall (Xu et al., 2022). MP stress resulted in the upregulation of organic metabolic pathways with concomitant alteration of MP mobility and absorption through the electrostatic and hydrophobic interactions between the root exudations and MPs (Xu et al., 2022). Nonetheless, the application of both negatively and positively charged PS MPs reduced Arabidopsis growth by altering gene expression (Sun et al., 2020).

The surface charge groups of MPs also determine the negative effects of MPs on root growth, for instance, functional groups such as -NH_2_ and -SO_3_H reduced root and shoot biomass and root volume (Xu et al., 2022). The -NH_2_ functional group contains a positive charge, which can easily occupy the binding sites and be easily adsorbed by the cell wall, thereby hindering the adsorption of cationic elements such as K^+^ Ca^2+^ or H^+^, Pb^2+^ around roots (Li et al., 2020; Song et al., 2023). Contrarily, -SO_3_H is of strong hydrophilic nature and contains a negative charge, and can closely combine with the hydrophobic functional group of the phospholipids in the cell membrane, thus it can easily enter the cells to cause cytotoxic effects in roots (Feng et al., 2019). Besides surface charge or functional group types, the zeta potential of MP particles also influences MP adsorption and translocation of MPs from soil to root (Hu et al., 2020). MPs also caused cell atrophy, increased conduct numbers, and lignification in roots (Xu et al., 2022). Lignification results in cell wall thickening and hardening, thereby reducing root activity (Rui et al., 2016; Singh et al., 2019). MPs with large surface areas adsorb essential elements around roots, thus reducing root growth (Abbasi et al., 2020). As mentioned above, MPs with different surface charge groups affect the kinetics of essential elements uptake and adsorption by roots. For example, functional groups such as -OH, -COOH, -SO_3_H, or -NH_2_ group might reduce elemental adsorption by blocking the extracellular adsorption pathway (Li et al., 2020a), by directly repelling elements with the same surface charges (Fu et al., 2022), or by the attraction and hetero-aggregation of opposite charge to each other, thereby reducing the element adsorption by roots (Li et al., 2021a; Xia et al., 2021; Song et al., 2023).

In addition to the aforementioned factors, different MPs cause different effects on root growth. For instance, PE MPs caused more negative effects on root weight, while PS MPs had more negative effects on root length (Shi et al., 2022). Moreover, Lian et al. (2022) showed that elliptical-shaped PE MPs have fewer negative effects on plant growth and bacterial communities in the soil as compared with sharp-edged PLA MPs. Such differential response of roots to different MPs might be due to (i) different shapes of MPs along with particle size, (ii) plant species-specificity to different MPs, and (iii) the toxicity level of different MPs after fragmentation and degradation.

**Figure 2:**
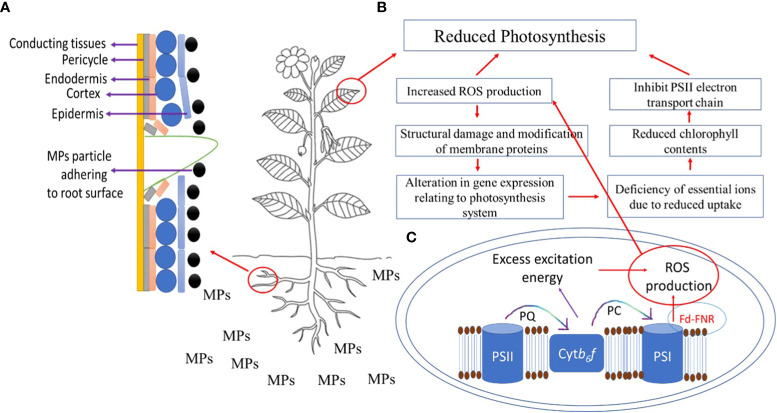
**(A)** Entry of microplastic particle via cracks on newly developed roots, **(B)** effects of microplastics (MPs) on photosynthesis, where QA-receptors (QA), photosystem II (PSII), photosystem I (PSI) plastocyanin (PC), cytochrome b6f complex (Ctyb*f*), Ferredoxin‐NADP^+^ reductase (FNR), ferredoxin (Fd), **(C)** ROS production in chloroplast under MPs stress.

MPs enter the ***shoot*** via xylem vessels as an action of transpiration pull and accumulate in leaves (Xu et al., 2022). After the accumulation of MPs in roots, they can enter the vasculature of the stems and leaves via the apoplastic pathway (Li et al., 2019); however, factors such as chemical composition and geometry of plastic debris, root surface area and volume, cell membrane potential, and xylem properties can influence the translocation of MPs from root to leaves via shoot. MPs after fragmentation can enter roots via cracks on newly developed roots (**Figure 2A**) and enter xylem or phloem vessels (Li et al., 2020). Thus, the likeliness of the entry of MPs through cracks in roots provides instant access to conducting tissues. MP stress negatively affects the growth of shoots and leaves (**Table 2**) by reducing cell elongation during the developmental phase, affecting the supply of nutrients, causing physical damage to xylem vessels, and influencing shoot and leaf biomass production. Furthermore, PS MP stress reduced leaves’ fresh weight, number of leaves per plant, leaf surface area, SPAD value, and overall plant height in a dose-dependent manner (Wang et al., 2023c). Liu et al. (2021) showed that PE MPs reduced shoot weight and shoot height significantly at high concentrations but stimulated root elongation, thus suggesting tissue-specific responses to MP stress.

***Plant yield*** refers to a plant’s end-of-life journey, and producing seeds is considered very important for maintaining a plant’s niche from the ecological point of view and for producing grains as a source of food for humans. MP stress significantly reduces plant yield by altering plant growth and development (Khalid et al., 2020). Nonetheless, intra-species responses among two rice cultivars (XS123 and Y900) were also observed in terms of rice yield under PS MP stress; such variation in rice yield was associated with the upregulation of the transcripts levels and accumulation of metabolites relating to energy expenditure pathways and metabolic accumulation pathways in XS123, thereby indicating the reprogramming of metabolites accumulation as a component of PS MP tolerance in XS123 (Wu et al., 2022). Likewise, Yi et al. (2023) showed that PE MP stress did not alter rice grain yield in a common cultivar, whereas hybrid rice showed a significant reduction in the number of grains per panicle (13%) and rice grain yield (23%). Such variation in rice yield was due to higher total amino acid contents in hybrid rice grain, whereas conventional rice grain did not show any difference in total amino acid content under PE MP stress and control (Yi et al., 2021). Moreover, the reduction in rice yield was ascribed to the MP stress-induced reduction in the hemoglobin content by directly destroying its tertiary structure and inhibiting root activity (Dong et al., 2022b). Likewise, the individual and combined application of PS MPs and Pb significantly reduced 100 seed weight and seed yield per plant, which was associated with the reduction in root weight, surface area, total amino acids, Rubisco activity, and impaired hormonal regulation (Chen et al., 2023a). Contrarily, exposure of rice to three types of MPs, including PET, PAN, and PE, did not cause any negative effects on rice yield, but rather increased rice yield and N contents in rice grains (Chen et al., 2022). In conclusion, MP stress-induced effects on yield are highly plant species-, cultivar-, and MP type-specific.

#### 4.1.2 Physiological growth reduction

MP-induced physical growth reduction is accompanied by the alteration in several key physiological mechanisms including photosynthesis, redox regulation, ionic homeostasis, and hormonal regulation. These mechanisms are sensitive to stressful conditions and any alteration in these mechanisms leads to crop growth reduction. In this section, we discuss the impacts of MPs on shoot and leaf physiology and root physiology.

***Effects on shoot and leaf physiology*:** Given the MP stress-induced reduction in shoot and leaf growth and biomass production, it is important to explore what physiological processes have been or could have been impacted by MP stress. ***Photosynthesis*** is an important mechanism in plants, which yields oxygen and energy in the form of sugars and depends on many factors including biosynthesis of photosynthetic pigments, chlorophyll fluorescence and leaf gas exchange, ionic homeostasis, and redox regulation. MP stress negatively regulates these factors, thus reducing photosynthesis in plants (Yang and Gao, 2022; **Figure 2B**). For instance, Lian et al. (2021) showed that the application of PS MPs reduced carotenoids and chlorophyll *a* and *b* by 12.5%, 9.1%, and 8.7% respectively, indicating one of the methods by which PS MP stress reduced shoot dry weight, shoot height, and leaf area in lettuce (*Lactuca sativa* L.). Likewise, in several other plant species, such as cucumber (*Cucumis sativus* L.), tomato, cabbage (*Brassica oleracea* L.), lettuce, and pakchoi (*Brassica rapa* L.), the application of PAN MPs showed a negative correlation with the chlorophyll *a* and *b* contents in leaves, thus reducing their growth (Ahammed et al., 2012). Liu et al. (2021) showed that the combined application of PAN with PE MPs significantly reduced photosynthesis by reducing the contents of chlorophyll *a* and *b* and carotenoids in a highly MP dose-dependent manner. In pumpkins, the application of PVC and PE MPs resulted in a dose-dependent reduction of chlorophyll contents and photosynthesis rate (Colzi et al., 2022). Gao et al. (2019) showed in lettuce that PE MP-induced reduction in photosynthesis was associated with a reduction in chlorophyll contents, leaf gas exchange, and Rubisco activity. Nonetheless, Mondal et al. (2022) observed a higher chlorophyll (a/b) ratio, probably because of the inhibition of chlorophyll ‘b’ synthesis under MP stress, suggesting the reduction in photosynthesis under MP stress could be due to less production of chlorophyll *b* (Pflugmacher et al., 2021). Conversely, Chai et al. (2023) showed that the PVC, PP, and PE MP-induced reduction in the photosynthesis of mangrove (*Kandelia obovate* L.) was due to a reduction in the concentration of carotenoids and chlorophyll *a*, and efficiency of ETC, while no significant effects of MP stress were observed on leaf gas exchange and chlorophyll *b* contents. These contrasting results can be explained by plant species-specificity and MP dose dependency. The plant species-specific response to MP stress was ascribed to the heterogenous responses of amino acids and hormones (Bouaicha et al., 2022).

Pollutants including MPs can disrupt the molecular structure of thylakoid membranes and can reduce the efficiency of ETC by inhibiting the activity of electron transfer in chloroplast, thus reducing photosynthesis. Though how MP stress induces these alterations remains elusive, this can be explained at least by the different surface functional groups of different MPs. MPs with -NH_2_ and -SO_3_H functional groups shut down the reaction center with a concomitant decrease in the number of election receptors in PSII (Kalhor et al., 2018), which results in reduced transfer of elections to QA-receptors in PSII (Wang et al., 2017), thereby reducing the overall efficiency of PSII under MP stress. Nonetheless, the kinetic curve of chlorophyll fluorescence was OJIP type, indicating the structure and function of the PSII photosystem were intact and unaltered after MP stress (Zha et al., 2021; Xu et al., 2022). MP stress reduced chlorophyll contents by converting the chlorophyll contents into phytol (Xu et al., 2022) and by inducing a high rate of the exfoliation of oxygen evolution complex (Wang et al., 2017; Xu et al., 2022), thus blocking the transfer of electrons to PSII reaction centers, which subsequently affects ETC reaction in the PSII photosystem. Reduction in photosynthesis was not only associated with a reduction in chlorophyll contents in the reaction center (Peng et al., 2021) but could also be associated with oxidation and reduction reactions and transfer of electrons in the reaction center of the PSII (Lu et al., 2020). Moreover, anthocyanins are very important pigments in plants, assisting in protecting leaves from photoinhibition by absorbing excess protons, thus also acting as non-enzymatic antioxidants. However, a reduction in anthocyanin biosynthesis also compromises photosynthesis under MP stress (Wang et al., 2023b).

Besides influencing the stomatal and non-stomatal limitations of photosynthesis, MP stress also reduces photosynthesis by affecting shoot and ***leaf ionome*** and causing the production of ROS (**Figure 2B**). Any alteration in uptake or accumulation of essential macro- and micro-nutrient in photosynthetically active tissues in shoots or leaves results in significant growth reduction. For instance, PP and PET MPs increased K concentration in shoots and leaves, while PVC MPs decreased K concentration thereby reducing photosynthetic efficiency (Colzi et al., 2022). Moreover, PE and PVC MPs increased nickel (Ni) concentration in shoots and leaves, which decreased after PP and PET MPs (Colzi et al., 2022). Reduction in photosynthesis, stomatal conductance, and SPAD values under PS and PVC MP stress were in line with the reduction in the concentration of Ca, K, N, and P in the shoot and leaves (Ma et al., 2022), nonetheless, these effects were highly MP type-specific and dose-dependent. Likewise, MP stress-induced reduction in N uptake may cause a low carbon fixation rate, and the disruption of chlorophyll contents or the decline in iron (Fe) contents in the shoot and leaves after exposure to PP- and PVC- MPs (Colzi et al., 2022) can partially inhibit the electron transport chain and lead to the loss of photosynthetic efficiency, and to the remodeling of the photosynthetic Fe-dependent protein and apparatus (Saito et al., 2014; Hantzis et al., 2018; Akmakjian et al., 2021). On the other hand, MPs such as PVC and PE increased Ni concentration in shoots and leaves, while PET and PP decreased Ni concentration in shoots and leaves (Colzi et al., 2022). Such imbalance in Ni concentration, either excessive uptake or deficiency, can reduce photosynthesis either by causing ROS production, in the case of excessive Ni concentration, or reduced N metabolism and reduced Fe uptake, in the case of Ni deficiency (Shahzad et al., 2018b).

The chloroplast is among the primary sites of ***ROS production*** during photosynthesis (**Figure 2C**), as the chloroplast converts light energy into energy required for chemical bonding. Under normal and optimal growth conditions, chlorophyll absorbs light and triggers several redox reactions in thylakoid membranes including oxidation of H_2_O, development of H^+^ gradient across thylakoid membranes, and reduction of NADP^+^ to NADPH (Khorobrykh et al., 2020). However, these reactions are highly sensitive to any abnormality experienced during any change in growth conditions, such as abiotic or biotic stress conditions (Krieger-Liszkay and Shimakawa, 2022). Exposure to different MPs resulted in a significantly high production of hydrogen peroxide (H_2_O_2_) (Pignattelli et al., 2021), one of the most important ROS, thereby reducing photosynthesis. However, Ren et al. (2018) found no difference in H_2_O_2_ production in MP-treated plants compared to control plants and this could be due to the pleiotropic effects of H_2_O_2_, or the activation of the antioxidant defense system and/or sensitivity of plant species to MP stress. Nonetheless, PS MPinduced higher ROS production may cause oxidative damage to the thylakoid membrane and chloroplast structure, thereby inhibiting photosynthesis (Lian et al., 2021). Moreover, MP stress reduced photochemical efficiency and caused higher production of H_2_O_2_ and thiobarbituric acid reactive substances, thereby contributing to a higher leaf loss rate under MP stress (Menicagli et al., 2022). It has also been suggested that MP stress might reduce the photosynthesis of C3 plants by inhibiting light use efficiency, while in C4 plants, reduction in photosynthesis could be mediated by the inhibition of carbon fixation (Liu et al., 2020; Zhang et al., 2023). Under such conditions, the stroma in the chloroplast may gather more electrons, which concomitantly may induce the production of ROS.

Photosynthesis is also regulated by ***several genes*** involved in chlorophyll biosynthesis, carbohydrate metabolism, and ATP production, and MP stress has also been shown to impact the regulation of such genes, thus reducing photosynthesis in plants. For instance, Liu et al. (2022) showed that PBAT-MP stress significantly affected photosynthesis in Arabidopsis (*Arabidopsis thaliana* L.) by downregulating the gene expression of genes that encode light-harvesting chlorophyll a/b binding (LHCB) proteins. Moreover, the downregulation of LHCB proteins affects plant stress adaptive responses by regulating redox homeostasis, and sensitivity of stomata to abscisic acid (Xu et al., 2012; Chen et al., 2023b), thereby suggesting PBAT MP stress-induced alteration in photosynthesis might relate to the ABA-mediated stomatal response (Liu et al., 2022). In tobacco (*Nicotiana tabacum* L.), the application of PE MPs resulted in the downregulation of more than 80% of differentially expressed genes relating to ETC, PSI, and PSII in chloroplast and light harvesting (Tang et al., 2022). Given the above discussion, it can be suggested that MP stress-induced reduction in photosynthesis (**Figure 2B**) results from six complementary mechanisms: (i) higher ROS production, leading to oxidative stress, (ii) structural damage and modification of membrane proteins in PSII, (iii) conversion of chlorophyll to phytol and plausibly higher activity of chlorophyllase enzyme due to higher ROS production, (iv) peroxidation of the chloroplast and thylakoid membrane, (v) alteration in leaf ionome, and (vi) alteration in gene expression relating to the photosynthesis system.

***Effects on root physiology:*** MP stress disrupts the balance between ***ROS production*** and ROS scavenging by increasing the ROS production beyond the capacity of plants to activate the antioxidant defense system, which subsequently disrupts the integrity of membranes, decreases mitotic cell division, and even causes cytogenetic anomalies and micronuclei (Giorgetti et al., 2020) in roots. MP stress significantly increases ROS (O_2_^-^ and H_2_O_2_) production in roots (Gao et al., 2019) and causes genotoxic effects (Jiang et al., 2019), thus reducing the number of rootlets (Li et al. (2021b). Nonetheless, Biba et al. (2023) observed no drastic changes in O_2_^-^ production in onion roots under different PS MP stress levels; however, when compared with the effects of PMMA MPs, PS MP treatment showed relevantly higher O_2_^-^ production, thereby indicating MP-type response of ROS production in the root. Several factors can support such results such as the time point of ROS measurement, upregulation of the antioxidant defense system, and innate ability of roots to encounter ROS toxicity. Contrarily, the application of PS MPs resulted in a significant reduction in root length due to higher ROS production and cyto-genotoxicity (Giorgetti et al., 2020; Maity et al., 2020). Such contrasting results can also be explained by the particle size of MPs, as MPs with a small particle size can easily be internalized into root tissues and cause oxidative stress when compared to MPs with a large particle size (Roy et al., 2023), therefore plant species, plant age, and the particle size of MPs should be considered when studying MP induced oxidative damage to plants.

**Figure 3:**
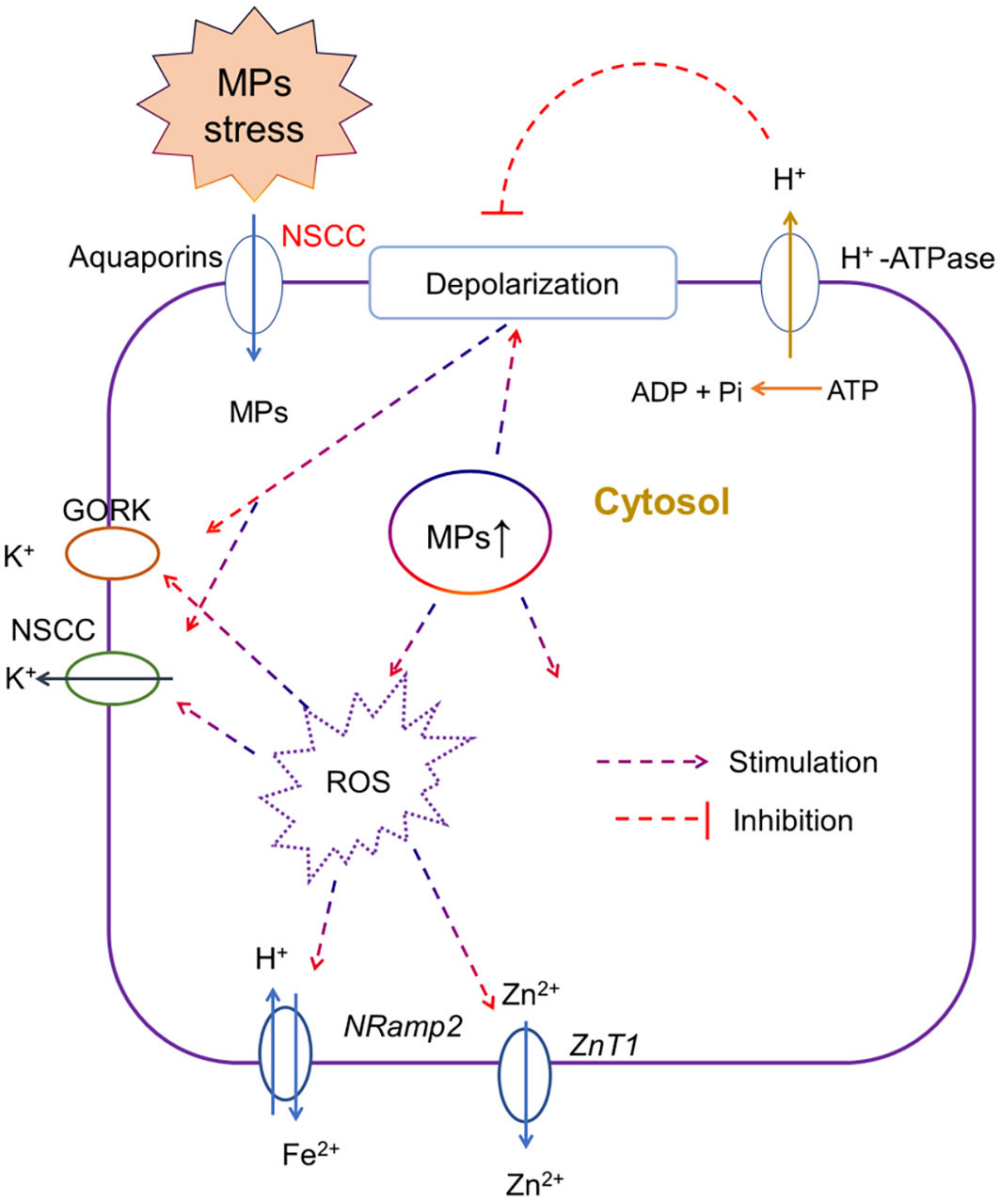
A putative model, representing the effects of microplastic (MP) stress on ionic homeostasis. Upon the entry of MP particles in roots via aquaporins or non-selective cation channels (NSCC), MPs may induce membrane depolarization and increase ROS production in the cytosol. Both membrane depolarization and excessive ROS production in the cytosol cause the activation of depolarization activated- and ROS activated- cation efflux channels, for instance, gated outwardly rectifying K^+^ channel (GORK) and NSCC for K^+^, *Natural resistance-associated macrophage proteins* (Nramps) channels for Fe^2+^ and Zinc transporter 1 (ZnT1) for Zn^2+^ at the plasma membrane, thereby decreasing the concentration of cations in the cytosol. This model needs to be validated in future studies using electrophysiological and pharmacological measurements.

Maintaining ***ionic homeostasis*** is very important for the activation of several adaptive responses including osmotic adjustment, water and nutrient uptake, and overall plant growth. Any disruption in ionic homeostasis especially under salt stress (Khan et al., 2020) or heavy metal stress disrupts ion transport (Zhao et al., 2019) via increasing membrane depolarization, ROS-activated cation efflux channels, and desensitization of ion channels (Huang et al., 2022). MP stress physically damages the roots, which affects the ionic homeostasis in plants. For instance, the exposure of lettuce to PS MP stress resulted in the downregulation of genes involved in ionic homeostasis, such as transition metal ions, cellular metal ions, and cellular manganese (Mn) ions in a dose-dependent manner (Wang et al., 2023c), thereby indicating that MP mediated disruption in ionic homeostasis resulted in hypertonic injury in lettuce roots. The foliar application of PS MPs resulted in a significant reduction in Fe, Mn, and Cu contents in roots as compared with the control, and given the essentiality of these elements in plant physiology (Lian et al., 2021), it is not surprising that the reduction in the accumulation of mineral nutrients is correlated with the overall root growth under MP stress. Moreover, reduction in elements such as Ni, Zn, or Fe also affects the antioxidant activity, and the biosynthesis of amino acids or specific proteins (Shahzad et al., 2018b; Colzi et al., 2022). Studies comparing the elemental profile of roots under MP stress showed that regardless of MP types and exposure time, MPs impaired root growth by reducing N, P (Shorobi et al., 2023), Fe (Briat et al., 2015), and K contents (Ma et al., 2022). However, Lian et al. (2019) showed that PVC MPs decreased Fe contents in the root, while PET MPs reduced Mg, Zn, and K, thereby suggesting the effects of MPs on ionic homeostasis might be MP type-specific. However, it is still unclear how MP stress affects ionic homeostasis in plants. Thus, we presented a model (**Figure 3**) and according to this model, we speculated that upon the entry of MP particles in roots, MPs induce membrane depolarization and increase ROS production in the cytosol. Both membrane depolarization and excessive ROS production in the cytosol cause the activation of depolarization activated- and ROS activated- cation (e.g., K, Ca, Fe, and Zn) efflux channels at the plasma membrane, thereby decreasing the concentration of cations in the cytosol. This model needs to be validated in future studies. Furthermore, Liu et al. (2023b) recently showed that PP MPs and rubber crumbs significantly increased ROS production and damaged the plasma membrane of root cells, as revealed by the decreased number of xylem vessels and reduced N uptake by roots. Thus, these results support our model.

MP stress also induces ***alterations in gene expression*** (Xu et al., 2020) in roots. As mentioned previously, MP stress induces cytotoxic and genotoxic effects on root growth, which might be due to an alteration in gene expression. For instance, Li et al. (2021b) showed that PAN MP treatment increased root aging or even death by affecting the expression of *CDC2* (a gene regulating the cell division cycle) and *CDK* (the coding gene of cyclin-dependent kinase). As both genes play a very crucial role in regulating the cell cycle, any alteration in the expression of these genes results in altered root growth and development. Application of PS MPs altered the overall root and plant growth by downregulating the genes involved in nitrogen metabolism and linolenic acid metabolism (Zhang et al., 2021), by altering the transcriptome and expression of genes encoding proteins involved in the tricarboxylic acid cycle (Wu et al., 2022) and accumulation of soluble protein (Yu et al., 2020). A recent study reported that PS MP stress-induced negative effects on root and shoot growth were associated with the downregulation of genes involved in ROS scavenging and ionic homeostasis (Wang et al., 2023c). Thus, MP stress affects root physiology by altering gene expression. However, a variation may exist among plant species, MP particle size, MP type, and other growth conditions, which needs to be further explored in future studies.

The activation of plant adaptive response to stress conditions is closely linked with the regulation of phytohormones (Tanveer et al., 2019; Huang et al., 2022), as ***hormonal regulation*** is very important in regulating several key physiological processes including ionic homeostasis, ROS scavenging, osmotic adjustment, and overall growth in plants. For instance, in wheat root, the application of PAN MP stress resulted in the upregulation of MiR164, which enhanced the silencing of NAC1 and weakened the association between auxin signaling and adventitious root initiation (Li et al., 2021b). Auxin and cytokinin govern the root system by regulating the formation of root hairs, crown roots, and lateral roots (Neogy et al., 2021). Li et al. (2021c) showed that PS MP stress reduces the number of rootlets and concentrations of three phytohormones including IBA, OPA, and cis-zeatin riboside in roots, thereby indicating PS MP stress affects the development of roots by changing cytokinin and auxin concentration. In rice, PS MP stress inhibited jasmonate and lignin contents by altering the expression of genes involved in their biosynthesis (Zhou et al., 2021). In conclusion, plant physical root growth is altered by five complementary mechanisms under MP stress, including ROS metabolism, altered gene expression, impaired ionic homeostasis, and hormonal regulation. Nonetheless, these mechanisms are highly plant species-specific, MP type-specific, particle size-specific, and dose-dependent.

### 4.2 Indirect effects

MP stress also causes several indirect effects on plants, which subsequently affect the plant root and shoot growth. For instance, MP stress alters soil bacterial community and reduces soil organic matter and bulk density. In this section, we have briefly overviewed how MPs induced indirect effects on plants by affecting soil fertility and productivity. Furthermore, as in-depth reviews relating to MP stress and its impact on soil fertility have recently been published (see Kumar et al., 2022; Tian et al., 2022; Wang et al., 2022; Shafea et al., 2023), we have briefly discussed the impact of MP stress on soil productivity to avoid any overlap with the abovementioned studies.

MPs in the soil system cause a series of negative effects on soil health and productivity, as MPs are dispersed in the soil through various processes, such as wet-dry cycles, bioturbation, harvesting, and soil management practices (Kumar et al., 2022). MPs cause significant effects on soil enzymatic activities. The application of fibrous PP decreased the activity of fluorescein diacetate hydrolase and urease in soil by 38% and 41%, respectively (Yi et al., 2021). Moreover, MPs have significant effects on the activities of CAT, PO, FDAase, and urease (Huang et al., 2019), which cause short-term changes in soil health. Soil bulk density is linked with soil porosity and the extent of the plant rooting system. It is also an important indicator of fertility (Wang et al., 2020b). Plastics usually have a lower density than soil minerals, thus altering soil bulk density and soil aeration. For instance, De Souza Machado et al. (2019) observed that soil bulk density was decreased in response to the addition of different MPs. Contrarily, Lozano et al. (2021) showed that PES and MFs enhanced soil aggregation due to better water retention and soil aeration. Lozano and Rillig (2020) confirmed that PES MFs can improve soil aeration, root penetration, and soil porosity, which ultimately improves plant growth. However, in maize (*Zea mays* L.), biodegradable MP residuals decreased soil bulk density, plant height, water-use efficiency (WUE), and grain yield as compared with PE MPs, thereby suggesting MP type-specific effects on grain yield (Uzamurera et al., 2023). Such discrepancy in the aforementioned effects that MPs have on soil bulk density can be explained in terms of the variation of soil composition and soil physical properties. Further research is required to explore the effects that MPs have on soil bulk density and plant root growth. MPs can also affect the water flow in soil, inducing drought due to increased evaporation, with subsequent effects on plant growth (Zhang et al., 2023). Moreover, changes in soil microbial activity due to MP stress also affect plant growth and nutrient uptake. For instance, LDPE MP stress changed the turnover of microbial communities in soil within 90 days (Wang et al., 2020b). PE MP stress changes the diversity of bacteria that are involved in nitrogen fixation in soil (Fei et al., 2020), which may result in the alterations of soil NH_4_^+^ and soil pH. Kalčíková et al. (2017) suggested that MP films and fibers can affect soil bacterial community more than the effects caused by different particle sizes, thus different factors affect the overall root growth and activity under MP stress. The nematode community showed reductive growth when exposed to LDPE MPs (Lin et al., 2020) and such decline in the nematode community could be due to several reasons such as ingestion of MPs and change of habitat caused by MP stress.

## 5 Targeting redox regulation to confer MP tolerance in plants

Higher plants require oxygen for energy production, thus the reduction of O_2_ into H_2_O results in the generation of different ROS, including hydroxyl radical (OH^-^), H_2_O_2_, and O_2_^-^. Different cellular compartments have the potential to become a source of ROS production such as the chloroplast or mitochondria. These ROS, at high concentrations, are highly cytotoxic in nature, thus their production and accumulation in different tissues must be tightly regulated and controlled (Tanveer and Shabala, 2018). MP stress increased ROS production, thus, to reinstate redox homeostasis, plants need to activate the antioxidant defense system. This system comprises numerous enzymatic and non-enzymatic antioxidants, however, the question is ‘does higher antioxidant activity mean higher stress tolerance?’. The answer can be described as ‘No’ due to two reasons: (i) ROS also act as signaling molecules and regulate several physiological mechanisms in plants, including cell expansion, systematically acquired resistance, acclimation, and hormonal regulation, and (ii) variation in the activation of different antioxidants at the tissue level and at different stress exposure times. This has provoked us to rethink the concept of ‘higher antioxidant-higher tolerance’ and include the signaling role of ROS in breeding programs to improve MP tolerance in plants. Some of the supporting arguments are discussed below.

### 5.1 Tissue specificity of antioxidant production

Different antioxidants showed differential activities in different tissues under MP stress. For instance, under PS MP stress a higher activation of GR and DHAR was observed only in roots, while only DHAR activity was increased in leaves, indicating the tissue specificity of the activation of the AsA-GSH cycle to encounter PS MP stress-induced oxidative stress (Li et al., 2021c). Likewise, Wang et al. (2023) showed that the activities of CAT, GR, and SOD increased in roots until day 6 of the application of MP stress, then tended to decline until day 12 after MP stress, and increased again on day 18. Whereas in leaves, the activities of SOD, GR, and APX were continuously increased from day 6 to day 18 in an MP dose-dependent manner. Likewise, CAT and SOD activities were completely inhibited under high PS MP concentration, while POD activity was not significantly affected under any PS MP concentration in rice roots (Zhang et al., 2021). Contrarily, in lettuce, there was a stronger activation of SOD (Zhang et al., 2023) and CAT (Yildiztugay et al., 2022) in leaves than in roots (Zhang et al., 2023), indicating a tissue-specific response of the antioxidant defense system to MP stress (Xu et al., 2021). Such varied responses of antioxidants in different tissues were also linked with the variation in ROS production in different tissues (Zhang et al., 2023), indicating that different tissues exhibited different antioxidant activities to counter ROS production under different MP stresses.

### 5.2 Temporal regulation of antioxidant production

The distinct activation of different antioxidants in different tissues also depends on the temporal production of ROS under MP stress. For instance, Liu et al. (2022) observed that the SOD enzyme activity was increased after 14 and 28 days of PBAT and LDPE MP stresses, while the POD activity was higher only after 14 and 28 days after PBAT MP stress, but POD activity first decreased after 14 days and then increased after 28 days of LDPE MP stress, which coincided with the higher rate of ROS production after 14 and 28 days of PBAT MP stress, and a higher rate of ROS production only after 28 days after LDPE MP stress, thereby indicating a temporal regulation of different antioxidants and ROS production under MP stress. Likewise, the higher production of H_2_O_2_ coincided with the higher activation of glutathione after 21 days of PVC and PE MP stresses (Pignattelli et al., 2020).

### 5.3 MP dose-dependent response of antioxidants

**Figure 4:**
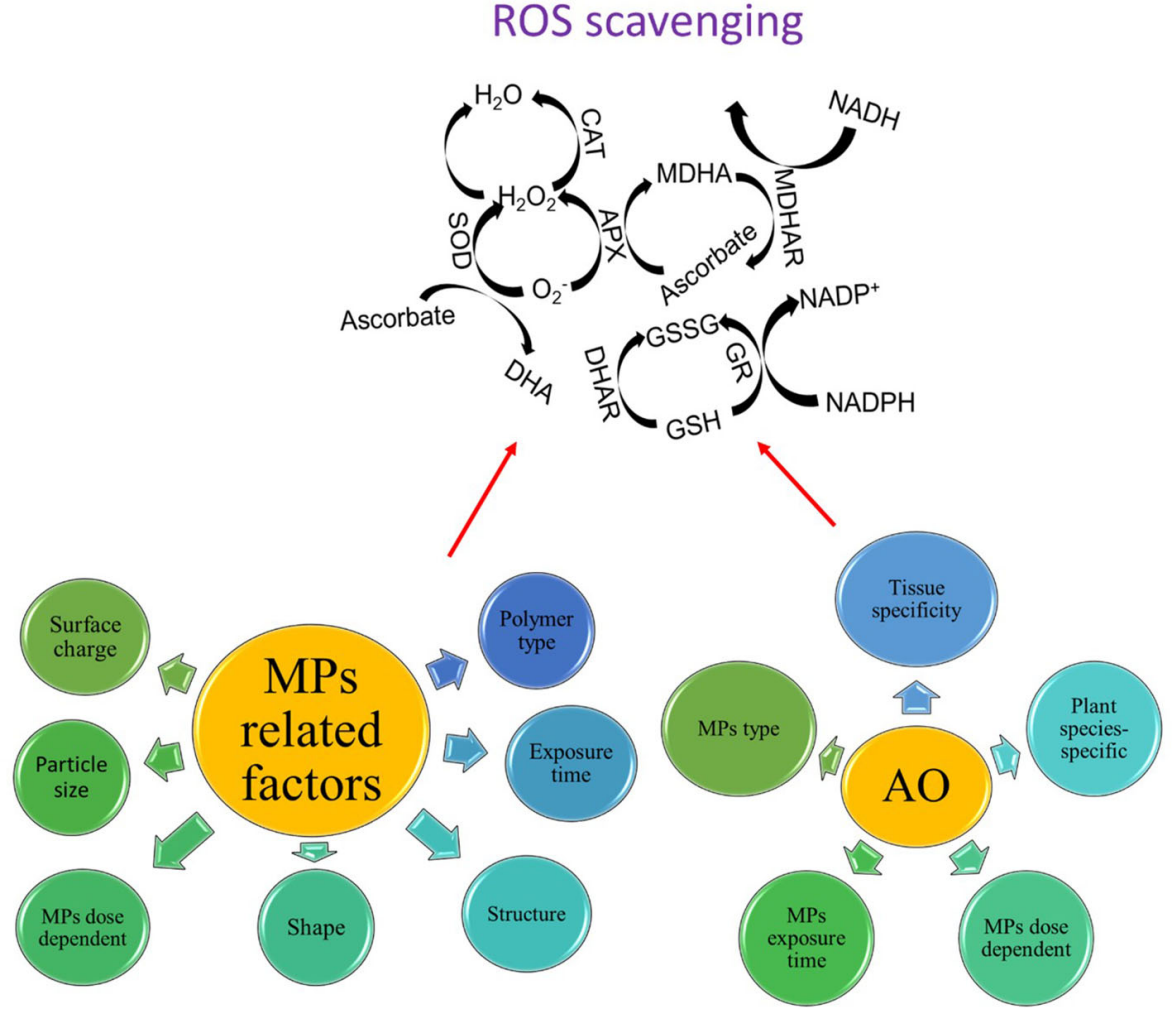
Different antioxidants (AO) work together in a highly orchestrated manner to scavenge ROS in response to MP stress, however, there are some MPs- and AO-related factors that may influence the overall efficiency of the ROS scavenging system.

The activation of antioxidants also varied in an MP dose-dependent manner. For instance, the activities of CAT, POD, and SOD increased when wheat roots were treated with 1% and 5% PE MPs, while the activities of CAT and SOD decreased in wheat roots treated with 8% PE MPs, thereby indicating that high concentrations of PE MPs exceeded the regulatory capacity of the antioxidant enzyme system (Liu et al., 2021). In rice, application of PS and PTFE MPs of less than 0.1 g L^-1^ resulted in relatively higher activation of CAT and SOD enzymes as compared with control, but when the dose of both MPs increased from 0.1 g L^-1^ to 0.2 g L^-1^, both enzymes showed a reductive activity as compared with control, thus suggesting the level of ROS production at 0.2 g L^-1^ concentration may exceed cell tolerance, subsequently inducing oxidative damage to cell and inhibiting/reducing the activities of antioxidants (Dong et al., 2020). Similar results were also reported in other plant species that had higher activation of different antioxidants with increasing MP concentration (Gao et al., 2019; Jiang et al., 2019; Liu et al., 2023a). Liu et al. (2023b) observed a dose-dependent SOD activity under PS MP stress while the activity of the POD enzyme was only higher at 10 mg L^-1^ PS MP stress. Likewise, POD and SOD activity increased only at 10% BP MP stress, while SOD activity was weakest at 1% BP MP stress (Sun et al., 2023). In conclusion, the activation of antioxidants is highly dose-dependent but not all antioxidants showed similar responses to dose-dependent effects of MP stress. Moreover, the abovementioned results also suggest that a temporary increase or activation of one or two antioxidant enzymes at a specific time point could be a regulatory response generated by the cell, while declined antioxidant activities could be due to the reason that ROS may already have consumed a large number of antioxidants, thus the accumulation of ROS may have exceeded the total antioxidant ability of cells under MP stress. Furthermore, the different antioxidant enzymes initiate different ROS scavenging reactions, for instance, the SOD enzyme is responsible for the dismutase of O_2_^-^ into H_2_O_2_, while CAT and GPX are responsible for converting H_2_O_2_ into H_2_O (Tanveer and Shabala, 2018). Thus, the different reaction activity of different antioxidants also determines the levels of overall ROS production under MP stress (**Figure 4**).

### 5.4 MP type-specific response of antioxidants

Different MP types also showed different regulation of antioxidant enzymes. For instance, SOD activity was relatively higher under 1% PE MP stress as compared with 1% BP stress, while BP MPs promoted more CAT activity than PE MPs (Sun et al., 2023). In tomatoes, PE and PS MP stresses were more detrimental in reducing antioxidants as compared with PP MP stress (Shi et al., 2022), indicating MPs with different polymer compositions may have different effects on the activation of antioxidants and plant growth. Shi et al. (2023) showed that the activation of antioxidants, leaf gas exchange, and chlorophyll contents were highly MP-type-specific. They found reduced SOD activity, no changes in POD activity, and enhanced CAT activity under all MP-type-specific stresses. MPs with different particle sizes induced different effects on the regulation of antioxidants in plants (Yu et al., 2022). For instance, Zhang et al. (2022) performed a meta-analysis and found that PVC and PP MPs caused neutral effects on the activities of antioxidants, while PS MPs caused 20% positive effects, PE MPs caused 31% positive effects, and PC MPs caused 100% positive effects on the activities of different antioxidants. PP and PVC MPs are commonly used in plastic covers and the phytotoxicity of these MPs may not come from these MPs themselves, but the reported phytotoxic effects may come from their leachates, such as chloride or toxic metal adsorbed on their surface, which promoted plant growth (Rozman and Kalčíková., 2022a). Nonetheless, this topic did not receive much attention, as factors such as particle size, surface functional group, and polymer composition of different MPs may regulate ROS scavenging differently.

### 5.5 Concluding remarks on targeting redox regulation to confer MP stress in plants

Antioxidants play a crucial role in reducing ROS production and governing plant growth under MP stress (**Figure 4**). MP stress-induced ROS production causes alterations in several metabolic processes both at extracellular and intracellular levels. Plants exhibit a very efficient ROS scavenging system to scavenge ROS, which is regarded as a redox regulatory mechanism and different antioxidants perform different ROS scavenging effects; nonetheless, these effects were highly tissue-specific, dose-dependent, and MP type-specific. A significant variation in the activation of different antioxidants was also observed after different exposure times of MP stress. Nonetheless, several reports indicated controversial results regarding the activation or not or the decrease or increase in the activation of different antioxidants under MP stress (see Sections 4.2 and 4.3). The possible reasons for such discrepancy can be ascribed to (i) the pleiotropic role of some ROS such as H_2_O_2_ in regulating adaptive response under MP stress, (ii) plant age-related differences in the activation of antioxidants in different cells and tissues, (iii) interspecific aspects of ROS scavenging vs ROS production, and (iv) the explicit role of some osmolytes including proline or glycine betaine as ROS scavenging agents, thereby replacing the need to activate more antioxidants and save energy to perform other metabolic processes under MP stress. Furthermore, the activation of antioxidants is an energy-consuming process and plants tend to consume available energy very deliberately to sustain their growth and photosynthesis, thus activating the antioxidant defense system may not be a reliable trait to confer MP tolerance in plants. Thus, it would not be highly beneficial to improve MP stress tolerance in plants by increasing the activities of some specific antioxidants using a marker-assisted selection (MAS) based approach or genetic engineering without considering temporal and tissue specificity of the MP stress-induced regulation of antioxidants. This also urges us to better understand and underpin the signaling role of ROS in regulating plant adaptive responses. The current literature analysis showed that there are some MP-related factors and antioxidant-related factors that may influence the overall activation of ROS scavenging in plants under MP stress (**Figure 4**). In this context, interactions such as MP particle size-ROS production-ROS scavenging system, MP composition-ROS production-ROS scavenging, and MP exposure time-ROS production-ROS scavenging should be considered as well.

## 6 Remediation methods for microplastic stress in agroecosystems

Different remediation and removal methods have been used globally for the removal of MPs in different environmental settings including agroecosystems and aquatic ecosystems (Kasmuri et al., 2022). Methods such as magnetic extraction, membrane bioreactors and filtration process, dynamic membrane technology, reverse osmosis and ultrafiltration, chemical degradation, and coagulant-based MP removal have different pros and cons and have different application directions in different industries. Moreover, biotechnological methods including metagenomic analysis, in silico mining, or protein engineering of enzymes have also been suggested for sustainable remediation of MP stress (Yadav et al., 2022). Moreover, the installation of micro-level filters in wastewater treatment plants can assist in reducing the MP contamination in terrestrial ecosystems including agroecosystems. Nonetheless, this requires extensive budgeting and developing countries may be not able to completely rely on such options, so despite the increasing awareness of MP pollution in soil and MP stress in plants, it is more important to emphasize the utilization of remediation methods based on the principles of agronomy in agroecosystems, especially in field crop production. Therefore, in this section, we have briefly discussed the potential role of different agronomic remediation methods in the context of MP stress.

### 6.1 Application of plant growth regulators

Plant growth regulators (PGRs) play a very critical role in conferring stress tolerance via the activation of stress-adaptive responses (Tanveer, 2019). However, MP stress reduces the endogenous levels of several PGRs, for example, declined endogenous contents of JA, SA, GA, and IAA under PS and PMMA (Ozfidan-Konakci et al., 2023) and ABA and JA under PS MP stress (Zhou et al., 2021). Nonetheless, the accumulation of PGRs in different tissues assists in coping with stress-induced adversities. For instance, increased contents of CK resulted in higher chlorophyll biosynthesis (Noein and Soleymani, 2022). Increment in SA resulted in higher tolerance by improving cellular permeability and N uptake (Zaid et al., 2019). Given the importance of different hormones in the regulation of plant growth, we only found three studies that showed the direct impact of the application of hormones on MP stress in plants.

Melatonin application reduced the translocation of nano-plastics from root to shoot by regulating the expression of genes, including the upregulation of TIP and PIP genes in roots and shoots (Li et al., 2021d). The study also found that PS NPs resulted in higher ROS production in roots and shoots with a concomitant reduction in chlorophyll contents and altered carbohydrate metabolism, while melatonin application activated the antioxidant defense system to reduce ROS production and confer MP tolerance in plants (Li et al., 2021d).

Brassinolides (also representing brassinosteroids) are stress-regulatory hormones that improve plant growth and confer stress tolerance in plants (Shahzad et al., 2018a; Tanveer et al., 2018; Tanveer et al., 2019). Recently, Gao et al. (2023) showed that brassinosteroid application resulted in reduced uptake of PS-NPs in tomatoes by regulating the expression of genes relating to aquaporins. Moreover, they also found that PS-NPs significantly reduced tomato growth, while brassinosteroid application improved tomato growth by enhancing amino acid and fatty acid metabolism and their synthesis (Gao et al., 2023).

Glutathione (GSH) is a very important member of the antioxidant defense system, and it, along with ascorbate, manages H_2_O_2_ accumulation and lipoxygenase activity. Recently, it has been shown that the negative effects of HDPE and PET MPs on the growth of rice were alleviated by the exogenous application of GSH (Chen et al., 2023c). The study found that both MPs significantly reduced chlorophyll contents and photosynthesis, while GSH application improved these physiological traits, thereby improving rice growth and yield (Chen et al., 2023c). This can partially be explained by the fact that GSH has also been distributed in mitochondria, where its higher accumulation leads to better ROS scavenging, thereby alleviating ROS-induced adverse effects on photosynthesis. Moreover, the higher accumulation of GSH in the cytosol can reduce the negative effects of ROS on the efflux of cations, including K^+^ and Ca^2+^, which support the findings of Chen et al. (2023c) relating to the improved uptake and accumulation of K, Ca, P, and Mg in roots and shoots with reduced ROS production under HDPE and PET MP stress.

### 6.2 Biochar application

For the effective removal of MPs, adsorbent porosity and surface area are two main factors that need to be considered (Siipola et al., 2020; Wang et al., 2022c), and biochar possesses both properties (Dad et al., 2020; Khan et al., 2022a; Kapoor et al., 2022). Biochar has great potential in ameliorating different soil-related abiotic stresses (Haider et al., 2022), including MP pollution in soil via increasing microbial activity, water restoration/retention, making complexes, and adsorption of MPs and heavy metals on biochar surface (Dad et al., 2020; Khan et al., 2022b; Ge et al., 2023). Nonetheless, the impact of biochar on the amelioration of MP-contaminated soil depends on the pyrolysis temperature (Makkawi et al., 2021; Palansooriya et al., 2022; Xiao et al., 2022) and the elemental composition of biochar feedstock (Ge et al., 2023). Furthermore, the application of biochar derived from sugar improved rice height, and rice yield under PS MP stress (Rassaei, 2023). Likewise, the application of corncob biochar was efficient in ameliorating the negative effects of PVC MPs on lettuce shoot growth (Li et al., 2023). Recently, it has been shown that date nuclei biochar improved root fresh and dry weight and overall root growth by increasing the mitotic index and reducing the percentage of abnormalities in root tip cells (Elbasiouny et al., 2023). Besides the direct impact of biochar on plant growth regulation under MP stress, the application of biochar has also been shown as an important strategy to improve soil productivity. For instance, biochar application improved the richness of soil bacterial community in MP-polluted soil and increased the abundance of genes regulating carbohydrate metabolism and amino acid metabolism, thereby facilitating N and P metabolism cycles in soil, and improving pepper plant growth in MP-polluted soil (Ran et al., 2023). Likewise, biochar application increased the activities of soil urease and dehydrogenase enzymes, soil organic matter, and bacterial/fungal community percentage and their abundance -16S rRNA genes/ITS in PVC polluted soil, thus improving wheat shoot growth and biomass (Khalid et al., 2023). Gao et al. (2022) showed that LDPE MPs significantly repressed the abundance of microorganisms and the expression of N and C cycling functional genes in the guts of earthworms, while biochar application elevated these effects in the earthworm, thereby indicating biochar application improves soil microbial activity. In conclusion, biochar application improves plant growth under MP stress by directly regulating plant growth and indirectly regulating soil fertility and productivity.

### 6.3 Biological degradation in soil

**Figure 5:**
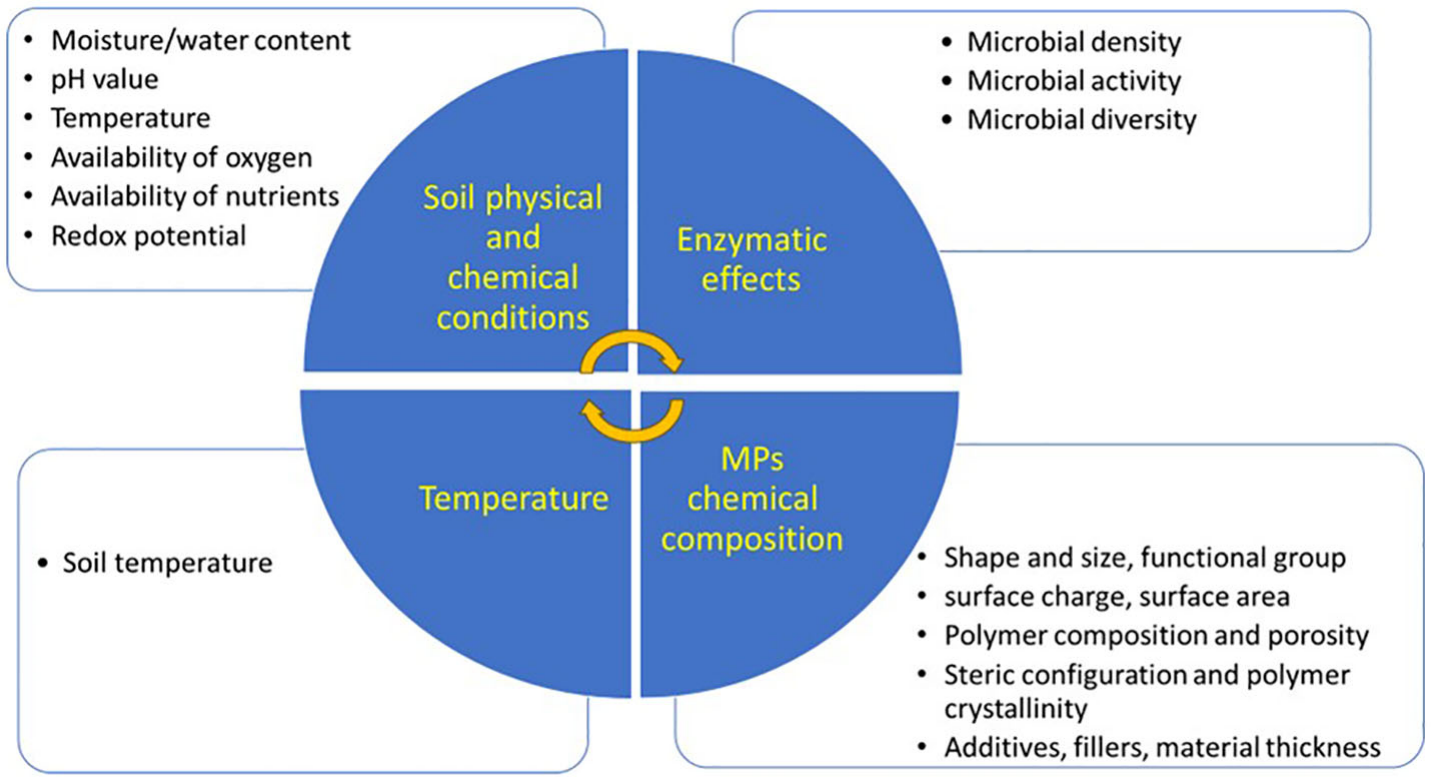
Factors influencing biodegradation of microplastics (MPs) in soil.

Microorganisms can degrade different hazardous materials in the environment (Shiu et al., 2020; Bhatt et al., 2019). MP polymers can be hydrolyzable due to the presence of microbial strains on them. These microbial strains produce enzymes that degrade the chemical structures of MP polymers (Bhatt et al., 2021). Microbial enzymes have catalytic potential due to the catalytic sites with amino acids, thus different strains of fungi and bacteria can induce the biodegradation of MPs. These microbial strains change the chemical structure of MPs from an oligomeric form to monomers (Ru et al., 2020). For instance, *Bacillus gottheilii* (a Bacillus bacteria) mediated the chemical alteration and bond cleavage, with subsequent reduction in the bioavailability of PS MPs (Auta et al., 2017). Likewise, Lysinibacillus species JJY0216 reduced the weight of PE and PP MPs by 9% and 4%, respectively, and produced various oxidation products containing CH_2_ groups during degradation (Jeon et al., 2021). Nitrogen-fixing bacteria degraded PBSA MPs by increasing the activities of plastic-degrading enzymes, and fungal abundance (Tanunchai et al., 2022). Nonetheless, several factors influence the efficiency of the biological degradation of MPs (**Figure 5**). The biological degradation by microorganisms works in two ways: intracellular degradation and extracellular degradation. In intracellular degradation, microbes accumulate on the surface of MPs to hydrolyze the MP plastics into short chains, while in extracellular degradation, bacteria produce extracellular enzymes, such as hydrolases, that help in the degradation of complex polymers into simpler units (Shah et al., 2008; Yuan et al., 2020). Through anaerobic or aerobic metabolism these short chains are converted by microorganisms into end-products, such as CO_2_, H_2_O, or CH_4_. The final consumption of these end-products is called biological natural attenuation (Tiwari et al., 2020). Thus, the biological degradation of MPs is ecologically a safe way to remove MPs from the environment Yang et al., 2021b.

### 6.4 Conservation agriculture

Conservation agriculture is a cropping system comprised of minimum soil disturbance, permanent soil cover, and crop diversification. The latter two aspects can play a very important role in ameliorating MP pollution in soil and MP stress in plants. For instance, the use of plant residues instead of plastic mulch can provide better water conservation in soil. It has been shown that MPs derived from the decomposition of plastic mulches reduce plant growth effectively; therefore, avoiding their usage may provide a better solution to reduce MP pollution in soil. Biodegradable plastic films are unlikely to create more problems than PE plastic films in terms of environmental damage (Sintim et al., 2019; Bandopadhyay et al., 2020). Adoption of crop diversification can also reduce the MP movement and pollution in soil. For instance, intercropping of halophytes with arable crops can be an effective approach, as several halophytes have been reported as excellent hyperaccumulators (Jiang et al., 2020; Jiang et al., 2021; Wang et al., 2021). For instance, the adherence of microbeads made of PE MPs, fibers, cellulose particles, and wood dust was examined on the surface of duckweed, and it was found that the adherence of PE MPs was far greater than other tested particles (Rozman and Kalčíková, 2022b). This can be explained by the initial interaction between duckweed and MPs, as negatively charged plant biomass attracts positively charged MPs (Kalčíková, 2020). Therefore, further studies are required to explore the scope of crop diversification and permanent soil cover in ameliorating MP pollution in soil and MP toxicity in plants.

## 7 Conclusion

**Figure 6:**
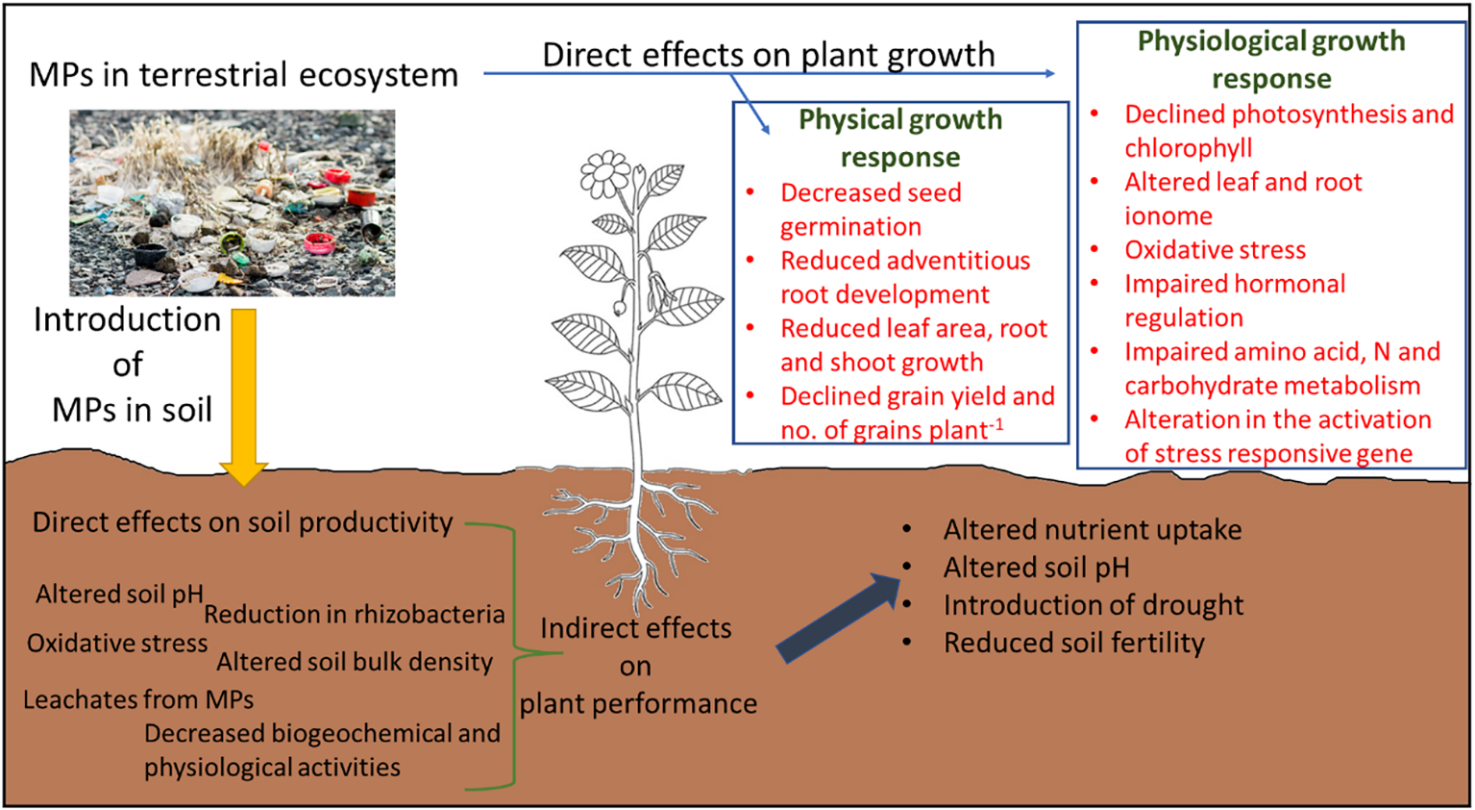
Graphical abstract summary of the direct and indirect impact of microplastic (MP) stress on plant growth and development.

The critical analysis of the literature showed that MP stress reduces plant growth and development by causing direct and indirect effects on plant growth (**Figure 6**), especially by regulating different physiological processes such as leaf and root ionome, redox homeostasis, hormonal regulation, photosynthesis, and energy dissipation. Nonetheless, these responses were highly tissue-, MP-type-specific, and dose-dependent. Moreover, the contrasting effects of MPs, of either growth stimulation or reduction, can also be explained by the microstructures of MPs, reactivity and crystallinity of MPs, chemical composition and additives added during fragmentation, and surface functional groups. Nevertheless, targeting redox regulation in plants can be a viable solution to improve MP stress in plants, but improving antioxidant activity might not improve MP tolerance in plants due to their complex and very time-specific response to different ROS. Thus, factors such as dose of MP, MP type, exposure time, and plant tissue specificity should also be considered in future breeding programs to improve MP tolerance in plants. However, this is a costly and time-consuming process, thus the adoption of agronomic techniques can also be useful under field conditions. We found that the use of crop residues such as live mulch instead of plastic mulch, the inclusion of halophytes for the phytoremediation of MPs, and the application of biochar and plant growth regulators alleviate MP stress in plants.

## Author contributions

Conceptualization, LH and MT; literature analysis, LJ, LL, YZ, and WF; writing-original draft preparation, XL, and QW; writing-review and editing, LJ, LL, LH, and MT. All authors contributed to the article and approved the submitted version.

## References

[B1] Abbasi S. Moore F. Keshavarzi B. Hopke P. K. Naidu R. Rahman M. M.. (2020). PET-microplastics as a vector for heavy metals in a simulated plant rhizosphere zone. Sci. Total Environ. 744, 140984. doi: 10.1016/j.scitotenv.2020.140984 32707415

[B2] Accinelli C. Abbas H. K. Bruno V. Khambhati V. H. Little N. S. Bellaloui N.. (2022). Field studies on the deterioration of microplastic films from ultra-thin compostable bags in soil. J. Environ. Manage. 305, 114407. doi: 10.1016/j.jenvman.2021.114407 34974218

[B3] Adeel M. Ma C. Ullah S. Rizwan M. Hao Y. Chen C.. (2019). Exposure to nickel oxide nanoparticles insinuates physiological, ultrastructural, and oxidative damage: A life cycle study on *Eisenia fetida* . Environ. Pollut. 254, 113032. doi: 10.1016/j.envpol.2019.113032 31454581

[B4] Adeel M. Shakoor N. Hussain T. Azeem I. Zhou P. Zhang P.. (2021). Bio-interaction of nano and bulk lanthanum and ytterbium oxides in soil system: Biochemical, genetic, and histopathological effects on *Eisenia fetida* . J. Hazardous Mater. 415, 125574. doi: 10.1016/j.jhazmat.2021.125574 33756203

[B5] Ahammed G. J. Wang M.-M. Zhou Y.-H. Xia X.-J. Mao W.-H. Shi K.. (2012). The growth, photosynthesis and antioxidant defense responses of five vegetable crops to phenanthrene stress. Ecotoxicol. Environ. Saf. 80, 132–139. doi: 10.1016/j.ecoenv.2012.02.015 22401953

[B6] Akmakjian G. Z. Riaz N. Guerinot M. L. (2021). Photoprotection during iron deficiency is mediated by the bHLH transcription factors PYE and ILR3. Proc. Natl. Acad. Sci. U.S.A. 118, e2024918118. doi: 10.1073/pnas.2024918118 34580211 PMC8501806

[B7] Alimi O. S. Farner Budarz J. Hernandez L. M. Tufenkji N. (2018). Microplastics and nanoplastics in aquatic environments: aggregation, deposition, and enhanced contaminant transport. Environ. Sci. Technol. 52 (4), 1704–1724. doi: 10.1021/acs.est.7b05559 29265806

[B8] Auta H. S. Emenike C. U. Fauziah S. H. (2017). Screening of Bacillus strains isolated from mangrove ecosystems in Peninsular Malaysia for microplastic degradation. Environ. Pollut. 231, 1552–1559. doi: 10.1016/j.envpol.2017.09.043 28964604

[B9] Azeem I. Adeel M. Ahmad M. A. Shakoor N. Jiangcuo G. D. Azeem K.. (2021). Uptake and accumulation of nano/microplastics in plants: a critical review. Nanomaterials 11 (11), 2935. doi: 10.3390/nano11112935 34835700 PMC8618759

[B10] Azeem I. Adeel M. Ahmad M. A. Shakoor N. Zain M. Yousef N.. (2022). Microplastic and nanoplastic interactions with plant species: trends, meta-analysis, and perspectives. Environ. Sci. Technol. Lett. 9 (6), 482–492. doi: 10.1021/acs.estlett.2c00107

[B11] Balestri E. Menicagli V. Ligorini V. Fulignati S. Galletti A. M.R. Lardicci C.. (2019). Phytotoxicity assessment of conventional and biodegradable plastic bags using seed germination test. Ecological Indicators 102, 569–580. doi: 10.1016/j.ecolind.2019.03.005

[B12] Bandopadhyay S. Sintim H. Y. DeBruyn J. M. (2020). Effects of biodegradable plastic film mulching on soil microbial communities in two agroecosystems. PeerJ 8, e9015. doi: 10.7717/peerj.9015 32341903 PMC7179572

[B13] Bao Y. Pan C. Li D. Guo A. Dai F. (2022). Stress response to oxytetracycline and microplastic-polyethylene in wheat (*Triticum aestivum* L.) during seed germination and seedling growth stages. Sci. Total Environ. 806, 150553. doi: 10.1016/j.scitotenv.2021.150553 34600215

[B14] Bhatt P. Huang Y. Zhan H. Chen S. (2019). Insight into microbial applications for the biodegradation of pyrethroid insecticides. Front. Microbiol. 10, 1778. doi: 10.3389/fmicb.2019.01778 31428072 PMC6687851

[B15] Bhatt P. Pathak V. M. Bagheri A. R. Bilal M. (2021). Microplastic contaminants in the aqueous environment, fate, toxicity consequences, and remediation strategies. Environ. Res. 200, 111762. doi: 10.1016/j.envres.2021.111762 34310963

[B16] Biba R. Cvjetko P. Jakopčić M. Komazec B. Tkalec M. Dimitrov N.. (2023). Phytotoxic effects of polystyrene and polymethyl methacrylate microplastics on *Allium cepa* roots. Plants 12 (4), 747. doi: 10.3390/plants12040747 36840096 PMC9959832

[B17] Bläsing M. Amelung W. (2018). Plastics in soil: Analytical methods and possible sources. Sci. Total Environ. 612, 422–435. doi: 10.1016/j.scitotenv.2017.08.086 28863373

[B18] Boots B. Russell C. W. Green D. S. (2019). Effects of microplastics in soil ecosystems: above and below ground. Environ. Sci. Technol. 53 (19), 11496–11506. doi: 10.1021/acs.est.9b03304 31509704

[B19] Bosker T. Bouwman L. J. Brun N. R. Behrens P. Vijver M. G. (2019). Microplastics accumulate on pores in seed capsule and delay germination and root growth of the terrestrial vascular plant *Lepidium sativum* . Chemosphere 226, 774–781. doi: 10.1016/j.chemosphere.2019.03.163 30965248

[B20] Bouaicha O. Tiziani R. Maver M. Lucini L. Miras-Moreno B. Zhang L.. (2022). Plant species-specific impact of polyethylene microspheres on seedling growth and the metabolome. Sci. Total Environ. 840, 156678. doi: 10.1016/j.scitotenv.2022.156678 35710005

[B21] Braun M. Mail M. Heyse R. Amelung W. (2021). Plastic in compost: Prevalence and potential input into agricultural and horticultural soils. Sci. Total Environ. 760, 143335. doi: 10.1016/j.scitotenv.2020.143335 33199003

[B22] Briat J.-F. Dubos C. Gaymard F. (2015). Iron nutrition, biomass production, and plant product quality. Trends Plant Sci. 20, 33–40. doi: 10.1016/j.tplants.2014.07.005 25153038

[B23] Campanale C. Galafassi S. Savino I. Massarelli C. Ancona V. Volta P.. (2022). Microplastics pollution in the terrestrial environments: Poorly known diffuse sources and implications for plants. Sci. Total Environ. 805, 150431. doi: 10.1016/j.scitotenv.2021.150431 34818779

[B24] Chai M. Li R. Li B. Wu H. Yu L. (2023). Responses of mangrove (*Kandelia obovate* L.) growth, photosynthesis, and rhizosphere soil properties to microplastic pollution. Mar. Pollut. Bull. 189, 114827. doi: 10.1016/j.marpolbul.2023.114827 36931158

[B25] Chen F. Aqeel M. Khalid N. Irshad M. K. Farhat F. Nazir A.. (2023c). Glutathione treatment suppresses the adverse effects of microplastics in rice. Chemosphere 322, 138079. doi: 10.1016/j.chemosphere.2023.138079 36775030

[B26] Chen F. Aqeel M. Khalid N. Nazir A. Irshad M. K. Akbar M. U.. (2023a). Interactive effects of polystyrene microplastics and Pb on growth and phytochemicals in mung bean (*Vigna radiata* L.). J. Hazardous Mater. 449, 130966. doi: 10.1016/j.jhazmat.2023.130966 36801714

[B27] Chen S. Feng Y. Han L. Li D. Feng Y. Jeyakumar P.. (2022). Responses of rice (*Oryza sativa* L.) plant growth, grain yield and quality, and soil properties to the microplastic occurrence in paddy soil. J. Soils Sediments 22, 2174–2183. doi: 10.1007/s11368-022-03232-w

[B28] Chen H. Jin J. Hu S. Shen L. Zhang P. Li Z.. (2023b). Metabolomics and proteomics reveal the toxicological mechanisms of florfenicol stress on wheat (*Triticum aestivum* L.) seedlings. J. Hazardous Mater. 443, 130264. doi: 10.1016/j.jhazmat.2022.130264 36327828

[B29] Chen Y. Leng Y. Liu X. Wang J. (2020). Microplastic pollution in vegetable farmlands of suburb Wuhan, central China. Environ. Pollut. 257, 113449. doi: 10.1016/j.envpol.2019.113449 31706776

[B30] Colzi I. Renna L. Bianchi E. Castellani M. B. Coppi A. Pignattelli S.. (2022). Impact of microplastics on growth, photosynthesis and essential elements in *Cucurbita pepo* L. J. Hazardous Mater. 423, 127238. doi: 10.1016/j.jhazmat.2021.127238 34844356

[B31] Corradini F. Meza P. Eguiluz R. Casado F. Huerta-Lwanga E. Geissen V. (2019). Evidence of microplastic accumulation in agricultural soils from sewage sludge disposal. Sci. Total Environ. 671, 411–420. doi: 10.1016/j.scitotenv.2019.03.368 30933797

[B32] Dad F. P. Khan W.-D. Tanveer M. Ramzani P. M. A. Shaukat R. Muktadir A. (2020). Influence of iron-enriched biochar on cd sorption, its ionic concentration and redox regulation of radish under cadmium toxicity. Agriculture 11, 1. doi: 10.3390/agriculture11010001

[B33] Delangiz N. Aliyar S. Pashapoor N. Nobaharan K. Lajayer B. A. Rodríguez-Couto S. (2022). Can polymer-degrading microorganisms solve the bottleneck of plastics’ environmental challenges? Chemosphere 294, 133709. doi: 10.1016/j.chemosphere.2022.133709 35074325

[B34] De Silva Y. S. K. Rajagopalan U. M. Kadono H. Li D. (2022). Effects of microplastics on lentil (Lens culinaris) seed germination and seedling growth. Chemosphere 303, 135162. doi: 10.1016/j.chemosphere.2022.135162 35654234

[B35] De Souza Machado A. A. Lau C. W. Kloas W. Bergmann J. Bachelier J. B. Faltin E.. (2019). Microplastics can change soil properties and affect plant performance. Environ. Sci. Technol. 53, 6044–6052. doi: 10.1021/acs.est.9b01339 31021077

[B36] Dong Y. Bao Q. Gao M. Qiu W. Song Z. (2022b). A novel mechanism study of microplastic and As co-contamination on indica rice (*Oryza sativa* L.). J. Hazardous Mater. 421, 126694. doi: 10.1016/j.jhazmat.2021.126694 34332483

[B37] Dong Y. Gao M. Song Z. Qiu W. (2020). Microplastic particles increase arsenic toxicity to rice seedlings. Environ. Pollut. 259, 113892. doi: 10.1016/j.envpol.2019.113892 31931412

[B38] Dong R. Liu R. Xu Y. Liu W. Wang L. Liang X.. (2022a). Single and joint toxicity of polymethyl methacrylate microplastics and As (V) on rapeseed (*Brassia campestris* L.). Chemosphere 291, 133066. doi: 10.1016/j.chemosphere.2021.133066 34861256

[B39] Duan J. Bolan N. Li Y. Ding S. Atugoda T. Vithanage M.. (2021). Weathering of microplastics and interaction with other coexisting constituents in terrestrial and aquatic environments. Water Res. 196, 117011. doi: 10.1016/j.watres.2021.117011 33743325

[B40] Elbasiouny H. Mostafa A. A. Zedan A. Elbltagy H. M. Dawoud S. F. Elbanna B. A.. (2023). Potential effect of biochar on soil properties, microbial activity and vicia faba properties affected by microplastics contamination. Agronomy 13 (1), 149. doi: 10.3390/agronomy13010149

[B41] Esterhuizen M. Kim Y. J. (2022). Effects of polypropylene, polyvinyl chloride, polyethylene terephthalate, polyurethane, high-density polyethylene, and polystyrene microplastic on *Nelumbo nucifera* (Lotus) in water and sediment. Environ. Sci. Pollut. Res. 29, 17580–17590. doi: 10.1007/s11356-021-17033-0 PMC8873133 34669136

[B42] Esterhuizen M. Vikfors S. Penttinen O.-P. Kim Y. J. Pflugmacher S. (2022). Lolium multiflorum germination and growth affected by virgin, naturally, and artificially aged high-density polyethylene microplastic and leachates. Front. Environ. Sci. 10. doi: 10.3389/fenvs.2022.964230

[B43] Fajardo C. Martín C. Costa G. Sánchez-Fortún S. Rodríguez C. De Lucas Burneo J. J.. (2022). Assessing the role of polyethylene microplastics as a vector for organic pollutants in soil: ecotoxicological and molecular approaches. Chemosphere 288, 132460. doi: 10.1016/j.chemosphere.2021.132460 34610374

[B44] Fei Y. Huang S. Zhang H. Tong Y. Wen D. Xia X.. (2020). Response of soil enzyme activities and bacterial communities to the accumulation of microplastics in an acid cropped soil. Sci. Total Environ. 707, 135634. doi: 10.1016/j.scitotenv.2019.135634 31761364

[B45] Feng L. J. Li J. W. Xu E. G. Sun X. D. Zhu F. P. Ding Z.. (2019). Short-term exposure to positively charged polystyrene nanoparticles causes oxidative stress and membrane destruction in cyanobacteria. Environ. Sci. Nano 6, 3072–3079. doi: 10.1039/C9EN00807A

[B46] Fu Q. Lai J. Ji X. Luo Z. Wu G. Luo X. (2022). Alterations of the rhizosphere soil microbial community composition and metabolite profiles of *Zea mays* by polyethylene-particles of different molecular weights. J. Hazardous Mater. 423, 127062. doi: 10.1016/j.jhazmat.2021.127062 34482080

[B47] Gao B. Li Y. Zheng N. Liu C. Ren H. Yao H. (2022). Interactive effects of microplastics, biochar, and earthworms on CO2 and N2O emissions and microbial functional genes in vegetable-growing soil. Environ. Res. 213, 113728. doi: 10.1016/j.envres.2022.113728 35732203

[B48] Gao M. Liu Y. Song Z. (2019). Effects of polyethylene microplastic on the phytotoxicity of di-n-butyl phthalate in lettuce (*Lactuca sativa* L. var. ramosa Hort). Chemosphere 237, 124482. doi: 10.1016/j.chemosphere.2019.124482 31398608

[B49] Gao M. Xu Y. Liu Y. Wang S. Wang C. Dong Y.. (2021). Effect of polystyrene on di-butyl phthalate (DBP) bioavailability and DBP-induced phytotoxicity in lettuce. Environ. Pollut. 268, 115870. 33120154 10.1016/j.envpol.2020.115870

[B50] Gao M. Wang Z. Jia Z. Zhang H. Wang T. (2023). Brassinosteroids alleviate nanoplastic toxicity in edible plants by activating antioxidant defense systems and suppressing nanoplastic uptake. Environ. Int. 174, 107901. 37003216 10.1016/j.envint.2023.107901

[B51] Ge J. Li H. Liu P. Zhang Z. Ouyang Z. Guo X. (2021). Review of the toxic effect of microplastics on terrestrial and aquatic plants. Sci. Total Environ. 791, 148333. doi: 10.1016/j.scitotenv.2021.148333 34412379

[B52] Ge S. Wang S. Mai W. Zhang K. Tanveer M. Wang L.. (2023). Characteristics and acidic soil amelioration effects of biochar derived from a typical halophyte Salicornia europaea L.(common glasswort). Environ. Sci. Pollut. Res. 30 (24), 66113–66124. doi: 10.1007/s11356-023-27182-z 37097582

[B53] Geyer R. Jambeck J. R. Law K. L. (2017). Production, use, and fate of all plastics ever made. Sci. Adv. 3, e1700782. doi: 10.1126/sciadv.1700782 28776036 PMC5517107

[B54] Giorgetti L. Spanò C. Muccifora S. Bottega S. Barbieri F. Bellani L.. (2020). Exploring the interaction between polystyrene nanoplastics and *Allium cepa* during germination: Internalization in root cells, induction of toxicity and oxidative stress. Plant Physiol. Biochem. 149, 170–177. doi: 10.1016/j.plaphy.2020.02.014 32070910

[B55] Gong W. Zhang W. Jiang M. Li S. Liang G. Bu Q.. (2021). Species-dependent response of food crops to polystyrene nanoplastics and microplastics. Sci. Total Environ. 796, 148750. doi: 10.1016/j.scitotenv.2021.148750 34265617

[B56] Guo X. Liu Y. Wang J. (2019). Sorption of sulfamethazine onto different types of microplastics: A combined experimental and molecular dynamics simulation study. Mar. Pollut. Bull. 145, 547–554. doi: 10.1016/j.marpolbul.2019.06.063 31590822

[B57] Guo M. Zhao F. Tian L. Ni K. Lu Y. Borah P. (2022). Effects of polystyrene microplastics on the seed germination of herbaceous ornamental plants. Sci. Total Environ. 809, 151100. doi: 10.1016/j.scitotenv.2021.151100 34695466

[B58] Haider F. U. Wang X. Farooq M. Hussain S. Cheema S. A. Ain N. U.. (2022). Biochar application for the remediation of trace metals in contaminated soils: Implications for stress tolerance and crop production. Ecotoxicol. Environ. Saf. 230, 113165. doi: 10.1016/j.ecoenv.2022.113165 34998263

[B59] Hantzis L. J. Kroh G. E. Jahn C. E. Cantrell M. Peers G. Pilon M.. (2018). A program for iron economy during deficiency targets specific fe proteins. Plant Physiol. 176, 596–610. doi: 10.1104/pp.17.01497 29150559 PMC5761800

[B60] Hernández-Arenas R. Beltrán-Sanahuja A. Navarro-Quirant P. Sanz-Lazaro C. (2021). The effect of sewage sludge containing microplastics on growth and fruit development of tomato plants. Environ. Pollut. 268, 115779. doi: 10.1016/j.envpol.2020.115779 33075680

[B61] Hu P. An J. Faulkner M. M. Wu H. Li Z. Tian X.. (2020). Nanoparticle charge and size control foliar delivery efficiency to plant cells and organelles. ACS Nano 14, 7970–7986. doi: 10.1021/acsnano.9b09178 32628442

[B62] Huang Y. Liu Q. Jia W. Yan C. Wang J. (2020). Agricultural plastic mulching as a source of microplastics in the terrestrial environment. Environ. Pollut. 260, 114096. doi: 10.1016/j.envpol.2020.114096 32041035

[B63] Huang X. Tanveer M. Min Y. Shabala S. (2022). Melatonin as a regulator of plant ionic homeostasis: implications for abiotic stress tolerance. J. Exp. Bot. 73, 5886–5902. doi: 10.1093/jxb/erac224 35640481

[B64] Huang D. Tao J. Cheng M. Deng R. Chen S. Yin L.. (2021). Microplastics and nanoplastics in the environment: Macroscopic transport and effects on creatures. J. Hazardous Mater. 407, 124399. doi: 10.1016/j.jhazmat.2020.124399 33191019

[B65] Huang Y. Zhao Y. Wang J. Zhang M. Jia W. Qin X. (2019). LDPE microplastic films alter microbial community composition and enzymatic activities in soil. Environ. Pollut. 254, 112983. doi: 10.1016/j.envpol.2019.112983 31394342

[B66] Hurley R. R. Nizzetto L. (2018). Fate and occurrence of micro(nano)plastics in soils: Knowledge gaps and possible risks. Curr. Opin. Environ. Sci. Health 1, 6–11. doi: 10.1016/j.coesh.2017.10.006

[B67] Jambeck J. R. Geyer R. Wilcox C. Siegler T. R. Perryman M. Andrady A.. (2015). Plastic waste inputs from land into the ocean. Science 347 (6223), 768–771. doi: 10.1126/science.1260352 25678662

[B68] Jeon J. M. Park S. J. Choi T. R. Park J. H. Yang Y. H. Yoon J. J. (2021). Biodegradation of polyethylene and polypropylene by *Lysinibacillus* species JJY0216 isolated from soil grove. Polymer Degradation Stability 191, 109662. doi: 10.1016/j.polymdegradstab.2021.109662

[B69] Jia H. Wu D. Yu Y. Han S. Sun L. Li M. (2022). Impact of microplastics on bioaccumulation of heavy metals in rape (Brassica napus L.). Chemosphere 288, 132576. 34656617 10.1016/j.chemosphere.2021.132576

[B70] Jiang X. Chen H. Liao Y. Ye Z. Li M. Klobučar G. (2019). Ecotoxicity and genotoxicity of polystyrene microplastics on higher plant *Vicia faba* . Environ. Pollut. 250, 831–838. doi: 10.1016/j.envpol.2019.04.055 31051394

[B71] Jiang L. Tanveer M. Han W. Tian C. Wang L. (2020). High and differential strontium tolerance in germinating dimorphic seeds of *Salicornia europaea* . Seed Sci. Technol. 48, 231–239. doi: 10.15258/sst.2020.48.2.10

[B72] Jiang L. Wu X. Zhao Z. Zhang K. Tanveer M. Wang L.. (2021). Luobuma (Apocynum) – Cash crops for saline lands. Ind. Crops Products 173, 114146. doi: 10.1016/j.indcrop.2021.114146

[B73] Kalčíková G. (2020). Aquatic vascular plants – A forgotten piece of nature in microplastic research. Environ. Pollut. 262, 114354. doi: 10.1016/j.envpol.2020.114354 32193083

[B74] Kalčíková G. Žgajnar Gotvajn A. Kladnik A. Jemec A. (2017). Impact of polyethylene microbeads on the floating freshwater plant duckweed *Lemna minor* . Environ. Pollut. 230, 1108–1115. doi: 10.1016/j.envpol.2017.07.050 28783918

[B75] Kalhor M. S. Aliniaeifard S. Seif M. Asayesh E. J. Bernard F. Hassani B.. (2018). Title: Enhanced salt tolerance and photosynthetic performance: Implication of γ-amino butyric acid application in salt-exposed lettuce (*Lactuca sativa* L.) plants. Plant Physiol. Biochem. 130, 157–172. doi: 10.1016/j.plaphy.2018.07.003 29990769

[B76] Kapoor A. Sharma R. Kumar A. Sepehya S. (2022). Biochar as a means to improve soil fertility and crop productivity: a review. J. Plant Nutr. 45, 2380–2388. doi: 10.1080/01904167.2022.2027980

[B77] Kasmuri N. Tarmizi N. A. A. Mojiri A. (2022). Occurrence, impact, toxicity, and degradation methods of microplastics in environment—a review. Environ. Sci. Pollut. Res. 29, 30820–30836. doi: 10.1007/s11356-021-18268-7 35091947

[B78] Khalid N. Aqeel M. Noman A. (2020). Microplastics could be a threat to plants in terrestrial systems directly or indirectly. Environ. Pollut. 267, 115653. doi: 10.1016/j.envpol.2020.115653 33254725

[B79] Khalid A. R. Shah T. Asad M. Ali A. Samee E. Adnan F.. (2023). Biochar alleviated the toxic effects of PVC microplastic in a soil-plant system by upregulating soil enzyme activities and microbial abundance. Environ. Pollut. 332, 121810. doi: 10.1016/j.envpol.2023.121810 37201571

[B80] Khan Z. Fan X. Khan M. N. Khan M. A. Zhang K. Fu Y.. (2022b). The toxicity of heavy metals and plant signaling facilitated by biochar application: Implications for stress mitigation and crop production. Chemosphere 308, 136466. doi: 10.1016/j.chemosphere.2022.136466 36122746

[B81] Khan M. A. Kumar S. Wang Q. Wang M. Fahad S. Nizamani M. M.. (2023). Influence of polyvinyl chloride microplastic on chromium uptake and toxicity in sweet potato. Ecotoxicol. Environ. Saf. 251, 114526. doi: 10.1016/j.ecoenv.2023.114526 36634477

[B82] Khan W.-D. Shaukat R. Farooq M. A. Ashraf M. N. Nadeem F. Tanveer M.. (2022). Iron-doped biochar regulated soil nickel adsorption, wheat growth, its physiology and elemental concentration under contrasting abiotic stresses. Sustainability 14, 7852. doi: 10.3390/su14137852

[B83] Khan WuD. Tanveer M. Shaukat R. Ali M. Pirdad F. (2020). “An overview of salinity tolerance mechanism in plants,” in Hasanuzzaman M. Tanveer M. Salt and Drought Stress Tolerance in Plants. Signaling and Communication in Plants (Cham: Springer). doi: 10.1007/978-3-030-40277-8_1

[B84] Khorobrykh S. Havurinne V. Mattila H. Tyystjärvi E. (2020). Oxygen and ROS in photosynthesis. Plants 9, 91. doi: 10.3390/plants9010091 31936893 PMC7020446

[B85] Kibria G. Nugegoda D. Haroon A. K. Y. (2022). “Microplastic Pollution and Contamination of Seafood (Including Fish, Sharks, Mussels, Oysters, Shrimps and Seaweeds): A Global Overview,” in Microplastic Pollution Emerging Contaminants and Associated Treatment Technologies. Ed. Hashmi M. Z. (Cham: Springer International Publishing), 277–322. doi: 10.1007/978-3-030-89220-3_14

[B86] Krieger-Liszkay A. Shimakawa G. (2022). Regulation of the generation of reactive oxygen species during photosynthetic electron transport. Biochem. Soc. Trans. 50, 1025–1034. doi: 10.1042/BST20211246 35437580

[B87] Kumar A. Mishra S. Pandey R. Yu Z. G. Kumar M. Khoo K. S.. (2022). Microplastics in terrestrial ecosystems: Un-ignorable impacts on soil characterizes, nutrient storage and its cycling. TrAC Trends Analytical Chem. 158, 116869. doi: 10.1016/j.trac.2022.116869

[B88] Kumar M. Xiong X. He M. Tsang D. C. W. Gupta J. Khan E.. (2020). Microplastics as pollutants in agricultural soils. Environ. Pollut. 265, 114980. doi: 10.1016/j.envpol.2020.114980 32544663

[B89] Law K. L. Narayan R. (2022). Reducing environmental plastic pollution by designing polymer materials for managed end-of-life. Nat. Rev. Mater. 7 (2), 104–116.

[B90] Lee T.-Y. Kim L. Kim D. An S. An Y.-J. (2022). Microplastics from shoe sole fragments cause oxidative stress in a plant (Vigna radiata) and impair soil environment. J. Hazardous Mater. 429, 128306. doi: 10.1016/j.jhazmat.2022.128306 35101758

[B91] Lian J. P. Wu J. Xiong H.. (2020). Impact of polystyrene nanoplastics (PSNPs) on seed germination and seedling growth of wheat (Triticum aestivum L.). J. Hazard Mater 385, 121620. 31744724 10.1016/j.jhazmat.2019.121620

[B92] Li X. Chen L. Mei Q. Dong B. Dai X. Ding G.. (2018). Microplastics in sewage sludge from the wastewater treatment plants in China. Water Res. 142, 75–85. doi: 10.1016/j.watres.2018.05.034 29859394

[B93] Li S. Guo J. Wang T. Gong L. Liu F. Brestic M.. (2021d). Melatonin reduces nanoplastic uptake, translocation, and toxicity in wheat. J. Pineal Res. 71 (3), e12761. doi: 10.1111/jpi.12761 34392562

[B94] Li C. Liu J. Wang D. Kong L. Wu Y. Zhou X.. (2021a). Electrostatic attraction of cationic pollutants by microplastics reduces their joint cytotoxicity. Chemosphere 282, 131121. doi: 10.1016/j.chemosphere.2021.131121 34470166

[B95] Li L. Luo Y. Li R. Zhou Q. Peijnenburg W. J. G. M. Yin N.. (2020a). Effective uptake of submicrometre plastics by crop plants via a crack-entry mode. Nat. Sustainability 3, 929–937. doi: 10.1038/s41893-020-0567-9

[B96] Li S. Wang T. Guo J. Dong Y. Wang Z. Gong L.. (2021c). Polystyrene microplastics disturb the redox homeostasis, carbohydrate metabolism and phytohormone regulatory network in barley. J. Hazardous Mater. 415, 125614. doi: 10.1016/j.jhazmat.2021.125614 33725553

[B97] Li W. Wufuer R. Duo J. Wang S. Luo Y. Zhang D.. (2020). Microplastics in agricultural soils: Extraction and characterization after different periods of polythene film mulching in an arid region. Sci. Total Environ. 749, 141420. doi: 10.1016/j.scitotenv.2020.141420 32836118

[B98] Li J. Yu Y. Chen X. Yu S. Cui M. Wang S.. (2023). Effects of biochar on the phytotoxicity of polyvinyl chloride microplastics. Plant Physiol. Biochem. 195, 228–237. doi: 10.1016/j.plaphy.2023.01.022 36645927

[B99] Li J. Zhang H. Zhu J. Shen Y. Zeng N. Liu S.. (2021b). Role of miR164 in the growth of wheat new adventitious roots exposed to phenanthrene. Environ. Pollut. 284, 117204. doi: 10.1016/j.envpol.2021.117204 33910135

[B100] Li L. Zhou Q. Yin N. Tu C. Luo Y. (2019). Uptake and accumulation of microplastics in an edible plant. Chin. Sci. Bull. 64, 928–934. doi: 10.1360/N972018-00845

[B101] Lian J. Liu W. Meng L. Wu J. Chao L. Zeb A.. (2021). Foliar-applied polystyrene nanoplastics (PSNPs) reduce the growth and nutritional quality of lettuce (*Lactuca sativa* L.). Environ. Pollut. 280, 116978. doi: 10.1016/j.envpol.2021.116978 33780844

[B102] Lian Y. Liu W. Shi R. Zeb A. Wang Q. Li J.. (2022). Effects of polyethylene and polylactic acid microplastics on plant growth and bacterial community in the soil. J. Hazardous Mater. 435, 129057. doi: 10.1016/j.jhazmat.2022.129057 35650727

[B103] Lian J. Shen M. Liu W. (2019). Effects of microplastics on wheat seed germination and seedling growth. J. Agro-Environment Sci. 38 (4), 737–745.

[B104] Liao Y.-C. Nazygul J. Li M. Wang X.-L. Jiang L.-J. (2019). Effects of microplastics on the growth, physiology, and biochemical characteristics of wheat (Triticum aestivum). Huan jing ke xue= Huanjing kexue 40 (10), 4661–4667. doi: 10.13227/j.hjkx.201903113 31854836

[B105] Lin D. Yang G. Dou P. Qian S. Zhao L. Yang Y.. (2020). Microplastics negatively affect soil fauna but stimulate microbial activity: insights from a field-based microplastic addition experiment. Proc. R. Soc B. 287, 20201268. doi: 10.1098/rspb.2020.1268 PMC7542786 32873207

[B106] Liu M. Lu S. Song Y. Lei L. Hu J. Lv W.. (2018). Microplastic and mesoplastic pollution in farmland soils in suburbs of Shanghai, China. Environ. Pollut. 242, 855–862. doi: 10.1016/j.envpol.2018.07.051 30036839

[B107] Liu J. Wang P. Wang Y. Zhang Y. Xu T. Zhang Y.. (2022). Negative effects of poly(butylene adipate-co-terephthalate) microplastics on Arabidopsis and its root-associated microbiome. J. Hazardous Mater. 437, 129294. doi: 10.1016/j.jhazmat.2022.129294 35728316

[B108] Liu S. Wang J. Zhu J. Wang J. Wang H. Zhan X. (2021). The joint toxicity of polyethylene microplastic and phenanthrene to wheat seedlings. Chemosphere 282, 130967. doi: 10.1016/j.chemosphere.2021.130967 34082309

[B109] Liu Y. Xu F. Ding L. Zhang G. Bai B. Han Y.. (2023b). Microplastics reduce nitrogen uptake in peanut plants by damaging root cells and impairing soil nitrogen cycling. J. Hazardous Mater. 443, 130384. doi: 10.1016/j.jhazmat.2022.130384 36444071

[B110] Liu Y. Yue L. Wang C. Zhu X. Wang Z. Xing B. (2020). Photosynthetic response mechanisms in typical C3 and C4 plants upon La_2_O_3_ nanoparticle exposure. Environ. Sci.: Nano 7, 81–92. doi: 10.1039/C9EN00992B

[B111] Liu Y. Zhang Q. Cui W. Duan Z. Wang F. (2019). Toxicity of polyethylene microplastics to seed germination of mung bean. Environ. Dev. 31 (5), 123–125.

[B112] Liu L. Zhou Y. Wang C. Liu H. Xie R. Wang L.. (2023a). Oxidative damage in roots of rice (Oryza sativa L.) seedlings exposed to microplastics or combined with cadmium. Bull. Environ. Contam Toxicol. 110, 15. doi: 10.1007/s00128-022-03659-4 36520278

[B113] López M. D. Toro M. T. Riveros G. Illanes M. Noriega F. Schoebitz M.. (2022). Brassica sprouts exposed to microplastics: Effects on phytochemical constituents. Sci. Total Environ. 823, 153796. doi: 10.1016/j.scitotenv.2022.153796 35150680

[B114] Lozano Y. M. Lehnert T. Linck L. T. Lehmann A. Rillig M. C. (2021). Microplastic shape, polymer type, and concentration affect soil properties and plant biomass. Front. Plant Sci. 12. doi: 10.3389/fpls.2021.616645 PMC7920964 33664758

[B115] Lozano Y. M. Rillig M. C. (2020). Effects of microplastic fibers and drought on plant communities. Environ. Sci. Technol. 54 (10), 6166–6173. doi: 10.1021/acs.est.0c01051 32289223 PMC7241422

[B116] Lu K. Shen D. Liu X. Dong S. Jing X. Wu W.. (2020). Uptake of iron oxide nanoparticles inhibits the photosynthesis of the wheat after foliar exposure. Chemosphere 259, 127445. doi: 10.1016/j.chemosphere.2020.127445 32593005

[B117] Ma J. Aqeel M. Khalid N. Nazir A. Alzuaibr F. M. Al-Mushhin A. A. M.. (2022). Effects of microplastics on growth and metabolism of rice (Oryza sativa L.). Chemosphere 307, 135749. doi: 10.1016/j.chemosphere.2022.135749 35863412

[B118] Maity S. Chatterjee A. Guchhait R. De S. Pramanick K. (2020). Cytogenotoxic potential of a hazardous material, polystyrene microparticles on Allium cepa L. J. Hazardous Mater. 385, 121560. doi: 10.1016/j.jhazmat.2019.121560 31732349

[B119] Maity S. Guchhait R. Sarkar M. B. Pramanick K. (2022). Occurrence and distribution of micro/nanoplastics in soils and their phytotoxic effects: A review. Plant Cell Environ. 45 (4), 1011–1028. doi: 10.1111/pce.14248 35060135

[B120] Makhdoumi P. Pirsaheb M. Amin A. A. Kianpour S. Hossini H. (2023). Microplastic pollution in table salt and sugar: Occurrence, qualification and quantification and risk assessment. J. Food Composition Anal. 119, 105261. doi: 10.1016/j.jfca.2023.105261

[B121] Makkawi Y. El Sayed Y. Lyra D.-A. Pour F. H. Khan M. Badrelzaman M. (2021). Assessment of the pyrolysis products from halophyte Salicornia bigelovii cultivated in a desert environment. Fuel 290, 119518. doi: 10.1016/j.fuel.2020.119518

[B122] Mao X. Xu Y. Cheng Z. Yang Y. Guan Z. Jiang L.. (2022). The impact of microplastic pollution on ecological environment: a review. Front. Biosci. (Landmark Ed) 27, 46. doi: 10.31083/j.fbl2702046 35226989

[B123] Meng F. Yang X. Riksen M. Geissen V. (2022). Effect of different polymers of microplastics on soil organic carbon and nitrogen – A mesocosm experiment. Environ. Res. 204, 111938. doi: 10.1016/j.envres.2021.111938 34478726

[B124] Menicagli V. Castiglione M. R. Balestri E. Giorgetti L. Bottega S. Sorce C.. (2022). Early evidence of the impacts of microplastic and nanoplastic pollution on the growth and physiology of the seagrass Cymodocea nodosa. Sci. Total Environ. 838, 156514. doi: 10.1016/j.scitotenv.2022.156514 35679937

[B125] Mondal N. K. Kundu S. Debnath P. Mondal A. Sen K. (2022). Effects of polyethylene terephthalate microplastic on germination, biochemistry and phytotoxicity of Cicer arietinum L. and cytotoxicity study on Allium cepa L. Environ. Toxicol. Pharmacol. 94, 103908. doi: 10.1016/j.etap.2022.103908 35709962

[B126] Nel A. E. Mädler L. Velegol D. Xia T. Hoek E. M. V. Somasundaran P.. (2009). Understanding biophysicochemical interactions at the nano–bio interface. Nat. Mater 8, 543–557. doi: 10.1038/nmat2442 19525947

[B127] Neogy A. Singh Z. Mushahary K. K. K. Yadav S. R. (2021). Dynamic cytokinin signaling and function of auxin in cytokinin responsive domains during rice crown root development. Plant Cell Rep. 40, 1367–1375. doi: 10.1007/s00299-020-02618-9 33047229

[B128] Noein B. Soleymani A. (2022). Corn (Zea mays L.) physiology and yield affected by plant growth regulators under drought stress. J. Plant Growth Regul. 41, 672–681. doi: 10.1007/s00344-021-10332-3

[B129] Ozfidan-Konakci C. Yildiztugay E. Arikan B. Alp-Turgut F. N. Turan M. Cavusoglu H.. (2023). Responses of individual and combined polystyrene and polymethyl methacrylate nanoplastics on hormonal content, fluorescence/photochemistry of chlorophylls and ROS scavenging capacity in Lemna minor under arsenic-induced oxidative stress. Free Radical Biol. Med. 196, 93–107. doi: 10.1016/j.freeradbiomed.2023.01.015 36657731

[B130] Palansooriya K. N. Sang M. K. Igalavithana A. D. Zhang M. Hou D. Oleszczuk P.. (2022). Biochar alters chemical and microbial properties of microplastic-contaminated soil. Environ. Res. 209, 112807. doi: 10.1016/j.envres.2022.112807 35093312

[B131] Pan Z. Liu Q. Jiang R. Li W. Sun X. Lin H.. (2021). Microplastic pollution and ecological risk assessment in an estuarine environment: The Dongshan Bay of China. Chemosphere 262, 127876. doi: 10.1016/j.chemosphere.2020.127876 32771704

[B132] Pan Z. Liu Q. Xu J. Li W. Lin H. (2022). Microplastic contamination in seafood from Dongshan Bay in southeastern China and its health risk implication for human consumption. Environ. Pollut. 303, 119163. doi: 10.1016/j.envpol.2022.119163 35305345

[B133] Peng J. Feng Y. Wang X. Li J. Xu G. Phonenasay S.. (2021). Effects of nitrogen application rate on the photosynthetic pigment, leaf fluorescence characteristics, and yield of indica hybrid rice and their interrelations. Sci. Rep. 11, 7485. doi: 10.1038/s41598-021-86858-z 33820934 PMC8021548

[B134] Pflugmacher S. Sulek A. Mader H. Heo J. Noh J. H. Penttinen O.-P.. (2020). The influence of new and artificial aged microplastic and leachates on the germination of lepidium sativum L. Plants 9, 339. doi: 10.3390/plants9030339 32156049 PMC7154828

[B135] Pflugmacher S. Tallinen S. Kim Y. J. Kim S. Esterhuizen M. (2021). Ageing affects microplastic toxicity over time: Effects of aged polycarbonate on germination, growth, and oxidative stress of Lepidium sativum. Sci. Total Environ. 790, 148166. doi: 10.1016/j.scitotenv.2021.148166 34091331

[B136] Pignattelli S. Broccoli A. Piccardo M. Terlizzi A. Renzi M. (2021). Effects of polyethylene terephthalate (PET) microplastics and acid rain on physiology and growth of Lepidium sativum. Environ. Pollut. 282, 116997. doi: 10.1016/j.envpol.2021.116997 33819777

[B137] Pignattelli S. Broccoli A. Renzi M. (2020). Physiological responses of garden cress (L. sativum) to different types of microplastics. Sci. Total Environ. 727, 138609. doi: 10.1016/j.scitotenv.2020.138609 32339829

[B138] Plastics Europe. (2019). Plastics — the facts 2019: An analysis of European plastics production, demand and waste data. PlasticsEurope.

[B139] Plastics Europe. (2020). Plastics europe Market Research Group (PEMRG) and Conversion Market & Strategy GmbH, Final Report. Available at: https://www.plasticseurope.org/en/resources/publications/4312-plastics-facts-2020 (Accessed 21 June 2023).

[B140] Qi Y. Yang X. Pelaez A. M. Lwanga E. H. Beriot N. Gertsen H.. (2018). Macro-and micro-plastics in soil-plant system: effects of plastic mulch film residues on wheat (*Triticum aestivum*) growth. Sci. Total Environ. 645, 1048–1056. doi: 10.1016/j.scitotenv.2018.07.229 30248830

[B141] Ran T. Li J. Liao H. Zhao Y. Yang G. Long J. (2023). Effects of biochar amendment on bacterial communities and their function predictions in a microplastic-contaminated Capsicum annuum L. soil. Environ. Technol. Innovation 31, 103174. doi: 10.1016/j.eti.2023.103174

[B142] Rassaei F. (2023). Methane emissions and rice yield in a paddy soil: the effect of biochar and polystyrene microplastics interaction. Paddy Water Environ. 21, 85–97. doi: 10.1007/s10333-022-00915-5

[B143] Ren X. Zhu J. Liu H. Xu X. Liang C. (2018). Response of antioxidative system in rice (Oryza sativa) leaves to simulated acid rain stress. Ecotoxicol. Environ. Saf. 148, 851–856. doi: 10.1016/j.ecoenv.2017.11.046

[B144] Rillig M. C. Leifheit E. Lehmann J. (2021). Microplastic effects on carbon cycling processes in soils. PloS Biol. 19, e3001130. doi: 10.1371/journal.pbio.3001130 33784293 PMC8009438

[B145] Roy T. Dey T. K. Jamal M. (2023). Microplastic/nanoplastic toxicity in plants: an imminent concern. Environ. Monit Assess. 195, 27. doi: 10.1007/s10661-022-10654-z PMC9589797 36279030

[B146] Rozman U. Kalčíková G. (2022a). Seeking for a perfect (non-spherical) microplastic particle–the most comprehensive review on microplastic laboratory research. J. Hazardous Mater. 424, 127529. doi: 10.1016/j.jhazmat.2021.127529 34736190

[B147] Rozman U. Kalčíková G. (2022b). The response of duckweed lemna minor to microplastics and its potential use as a bioindicator of microplastic pollution. Plants 11 (21), 2953. doi: 10.3390/plants11212953 36365405 PMC9658923

[B148] Ru J. Huo Y. Yang Y. (2020). Microbial degradation and valorization of plastic wastes. Front. Microbiol. 11. doi: 10.3389/fmicb.2020.00442 PMC7186362 32373075

[B149] Rui H. Chen C. Zhang X. Shen Z. Zhang F. (2016). Cd-induced oxidative stress and lignification in the roots of two Vicia sativa L. varieties with different Cd tolerances. J. Hazardous Mater. 301, 304–313. doi: 10.1016/j.jhazmat.2015.08.052 26372696

[B150] Sahasa R. G. K. Dhevagi P. Poornima R. Ramya A. Moorthy P. S. Alagirisamy B.. (2023). Effect of polyethylene microplastics on seed germination of Blackgram (Vigna mungo L.) and Tomato (Solanum lycopersicum L.). Environ. Adv. 11, 100349. doi: 10.1016/j.envadv.2023.100349

[B151] Saito A. Shimizu M. Nakamura H. Maeno S. Katase R. Miwa E.. (2014). Fe deficiency induces phosphorylation and translocation of Lhcb1 in barley thylakoid membranes. FEBS Lett. 588, 2042–2048. doi: 10.1016/j.febslet.2014.04.031 24815689

[B152] Schiavo S. Oliviero M. Chiavarini S. Manzo S. (2020). Adverse effects of oxo-degradable plastic leachates in freshwater environment. Environ. Sci. Pollut. Res. 27, 8586–8595. doi: 10.1007/s11356-019-07466-z 31904098

[B153] Shafea L. Yap J. Beriot N. Felde V. J. M. N. L. Okoffo E. D. Enyoh C. E.. (2023). Microplastics in agroecosystems: A review of effects on soil biota and key soil functions. J. Plant Nutr. Soil Sci. 186, 5–22. doi: 10.1002/jpln.202200136

[B154] Shah A. A. Hasan F. Hameed A. Ahmed S. (2008). Biological degradation of plastics: a comprehensive review. Biotechnol. Adv. 26 (3), 246–265. doi: 10.1016/j.biotechadv.2007.12.005 18337047

[B155] Shahzad B. Tanveer M. Che Z. Rehman A. Cheema S. A. Sharma A.. (2018a). Role of 24-epibrassinolide (EBL) in mediating heavy metal and pesticide induced oxidative stress in plants: A review. Ecotoxicol. Environ. Saf. 147, 935–944. doi: 10.1016/j.ecoenv.2017.09.066 29029379

[B156] Shahzad B. Tanveer M. Rehman A. Cheema S. A. Fahad S. Rehman S.. (2018b). Nickel; whether toxic or essential for plants and environment - A review. Plant Physiol. Biochem. 132, 641–651. doi: 10.1016/j.plaphy.2018.10.014 30340176

[B157] Shi R. Liu W. Lian Y. Wang Q. Zeb A. Tang J. (2022). Phytotoxicity of polystyrene, polyethylene and polypropylene microplastics on tomato (Lycopersicon esculentum L.). J. Environ. Manage. 317, 115441. doi: 10.1016/j.jenvman.2022.115441 35661879

[B158] Shi R. Liu W. Lian Y. Zeb A. Wang Q. (2023). Type-dependent effects of microplastics on tomato (Lycopersicon esculentum L.): Focus on root exudates and metabolic reprogramming. Sci. Total Environ. 859, 160025. doi: 10.1016/j.scitotenv.2022.160025 36356752

[B159] Shiu R.-F. Vazquez C. I. Chiang C.-Y. Chiu M.-H. Chen C.-S. Ni C.-W.. (2020). Nano- and microplastics trigger secretion of protein-rich extracellular polymeric substances from phytoplankton. Sci. Total Environ. 748, 141469. doi: 10.1016/j.scitotenv.2020.141469 33113698

[B160] Shorobi F. M. Vyavahare G. D. Seok Y. J. Park J. H. (2023). Effect of polypropylene microplastics on seed germination and nutrient uptake of tomato and cherry tomato plants. Chemosphere 329, 138679. doi: 10.1016/j.chemosphere.2023.138679 37059201

[B161] Siipola V. Pflugmacher S. Romar H. Wendling L. Koukkari P. (2020). Low-cost biochar adsorbents for water purification including microplastics removal. App Sci. 10 (3), 788.

[B162] Singh V. Sergeeva L. Ligterink W. Aloni R. Zemach H. Doron-Faigenboim A.. (2019). Gibberellin promotes sweetpotato root vascular lignification and reduces storage-root formation. Front. Plant Sci. 10. doi: 10.3389/fpls.2019.01320 PMC6897044 31849998

[B163] Sintim H. Y. Bary A. I. Hayes D. G. English M. E. Schaeffer S. M. Miles C. A.. (2019). Release of micro- and nanoparticles from biodegradable plastic during in *situ* composting. Sci. Total Environ. 675, 686–693. doi: 10.1016/j.scitotenv.2019.04.179 31039503

[B164] Song Z. Zhao X. Dong Y. Bai L. Wang S. Gao M. (2023). Effects of polystyrene nanoplastics with different functional groups on the accumulation and toxicity of Pb on dandelion. Chemosphere 310, 136874. doi: 10.1016/j.chemosphere.2022.136874 36270525

[B165] Spanò C. Muccifora S. Castiglione M. R. Bellani L. Bottega S. Giorgetti L. (2022). Polystyrene nanoplastics affect seed germination, cell biology and physiology of rice seedlings in-short term treatments: Evidence of their internalization and translocation. Plant Physiol. Biochem. 172, 158–166. doi: 10.1016/j.plaphy.2022.01.012 35074726

[B166] Sridharan S. Kumar M. Saha M. Kirkham M. B. Singh L. Bolan N. S. (2022). The polymers and their additives in particulate plastics: What makes them hazardous to the fauna? Sci. Total Environ. 824, 153828. doi: 10.1016/j.scitotenv.2022.153828 35157873

[B167] Sun H. Shi Y. Zhao P. Long G. Li C. Wang J.. (2023). Effects of polyethylene and biodegradable microplastics on photosynthesis, antioxidant defense systems, and arsenic accumulation in maize (Zea mays L.) seedlings grown in arsenic-contaminated soils. Sci. Total Environ. 868, 161557. doi: 10.1016/j.scitotenv.2023.161557 36640877

[B168] Sun X.-D. Yuan X.-Z. Jia Y. Feng L.-J. Zhu F.-P. Dong S.-S.. (2020). Differentially charged nanoplastics demonstrate distinct accumulation in *Arabidopsis thaliana* . Nat. Nanotechnol. 15, 755–760. doi: 10.1038/s41565-020-0707-4 32572228

[B169] Tang M. Huang Y. Zhang W. Fu T. Zeng T. Huang Y.. (2022). Effects of microplastics on the mineral elements absorption and accumulation in hydroponic rice seedlings (Oryza sativa L.). Bull. Environ. Contam Toxicol. 108, 949–955. doi: 10.1007/s00128-021-03453-8 35079849

[B170] Tanunchai B. Kalkhof S. Guliyev V. Wahdan S. F. M. Krstic D. Schädler M.. (2022). Nitrogen fixing bacteria facilitate microbial biodegradation of a bio-based and biodegradable plastic in soils under ambient and future climatic conditions. Environ. Sci.: Processes Impacts 24 (2), 233–241. doi: 10.1039/d1em00426c 35048922

[B171] Tanveer M. (2019). Role of 24-epibrassinolide in inducing thermo-tolerance in plants. J. Plant Growth Regul. 38, 945–955. doi: 10.1007/s00344-018-9904-x

[B172] Tanveer M. Shabala S. (2018). “Targeting Redox Regulatory Mechanisms for Salinity Stress Tolerance in Crops,” in Salinity Responses and Tolerance in Plants, vol. 1. Eds. Kumar V. Wani S. H. Suprasanna P. Tran L.-S. P. (Cham: Springer International Publishing), 213–234. doi: 10.1007/978-3-319-75671-4_8

[B173] Tanveer M. Shahzad B. Sharma A. Biju S. Bhardwaj R. (2018). 24-Epibrassinolide; an active brassinolide and its role in salt stress tolerance in plants: A review. Plant Physiol. Biochem. 130, 69–79. doi: 10.1016/j.plaphy.2018.06.035 29966934

[B174] Tanveer M. Shahzad B. Sharma A. Khan E. A. (2019). 24-Epibrassinolide application in plants: An implication for improving drought stress tolerance in plants. Plant Physiol. Biochem. 135, 295–303. doi: 10.1016/j.plaphy.2018.12.013 30599306

[B175] Tian L. Jinjin C. Ji R. Ma Y. Yu X. (2022). Microplastics in agricultural soils: sources, effects, and their fate. Curr. Opin. Environ. Sci. Health 25, 100311. doi: 10.1016/j.coesh.2021.100311

[B176] Tiwari N. Santhiya D. Sharma J. G. (2020). Microbial remediation of micro-nano plastics: current knowledge and future trends. Environ. Pollut. 265, 115044. doi: 10.1016/j.envpol.2020.115044 32806397

[B177] Udovicki B. Andjelkovic M. Cirkovic-Velickovic T. Rajkovic A. (2022). Microplastics in food: scoping review on health effects, occurrence, and human exposure. Int. J. Food Contam. 9, 7. doi: 10.1186/s40550-022-00093-6

[B178] Urbina M. A. Correa F. Aburto F. Ferrio J. P. (2020). Adsorption of polyethylene microbeads and physiological effects on hydroponic maize. Sci. Total Environ. 741, 140216. doi: 10.1016/j.scitotenv.2020.140216 32886998

[B179] Uzamurera A. G. Wang P.-Y. Zhao Z.-Y. Tao X.-P. Zhou R. Wang W.-Y.. (2023). Thickness-dependent release of microplastics and phthalic acid esters from polythene and biodegradable residual films in agricultural soils and its related productivity effects. J. Hazardous Mater. 448, 130897. doi: 10.1016/j.jhazmat.2023.130897 36736218

[B180] van den Berg P. Huerta-Lwanga E. Corradini F. Geissen V. (2020). Sewage sludge application as a vehicle for microplastics in eastern Spanish agricultural soils. Environ. Pollut. 261, 114198. doi: 10.1016/j.envpol.2020.114198 32097788

[B181] Viljoen S. J. Brailsford F. L. Murphy D. V. Hoyle F. C. Chadwick D. R. Jones D. L. (2023). Leaching of phthalate acid esters from plastic mulch films and their degradation in response to UV irradiation and contrasting soil conditions. J. Hazardous Mater. 443, 130256. doi: 10.1016/j.jhazmat.2022.130256 36327845

[B182] Wang Y. Bai J. Wen L. Wang W. Zhang L. Liu Z.. (2023b). Phytotoxicity of microplastics to the floating plant Spirodela polyrhiza (L.): Plant functional traits and metabolomics. Environ. Pollut. 322, 121199. doi: 10.1016/j.envpol.2023.121199 36738884

[B183] Wang J. Huang M. Wang Q. Sun Y. Zhao Y. Huang Y. (2020b). LDPE microplastics significantly alter the temporal turnover of soil microbial communities. Sci. Total Environ. 726, 138682. doi: 10.1016/j.scitotenv.2020.138682 32481223

[B184] Wang J. Li J. Liu W. Zeb A. Wang Q. Zheng Z.. (2023a). Three typical microplastics affect the germination and growth of amaranth (Amaranthus mangostanus L.) seedlings. Plant Physiol. Biochem. 194, 589–599. doi: 10.1016/j.plaphy.2022.12.007 36529009

[B185] Wang Z. Sedighi M. Lea-Langton A. (2020c). Filtration of microplastic spheres by biochar: removal efficiency and immobilisation mechanisms. Water Res. 184, 116165. doi: 10.1016/j.watres.2020.116165 32688153

[B186] Wang L. Tanveer M. (2023). Editorial to the special issue “Eco-physiological and molecular basis of stress tolerance in plants.” Biology 12, 485. doi: 10.3390/biology12030485 36979176 PMC10045121

[B187] Wang F. Wang Q. Adams C. A. Sun Y. Zhang S. (2022). Effects of microplastics on soil properties: Current knowledge and future perspectives. J. Hazardous Mater. 424, 127531. doi: 10.1016/j.jhazmat.2021.127531 34740160

[B188] Wang L. Wang X. Jiang L. Zhang K. Tanveer M. Tian C.. (2021). Reclamation of saline soil by planting annual euhalophyte Suaeda salsa with drip irrigation: A three-year field experiment in arid northwestern China. Ecol. Eng. 159, 106090. doi: 10.1016/j.ecoleng.2020.106090

[B189] Wang Y. Xiang L. Wang F. Redmile-Gordon M. Bian Y. Wang Z.. (2023c). Transcriptomic and metabolomic changes in lettuce triggered by microplastics-stress. Environ. Pollut. 320, 121081. doi: 10.1016/j.envpol.2023.121081 36646407

[B190] Wang Y. Xu C. Wu M. Chen G. (2017). Characterization of photosynthetic performance during reproductive stage in high-yield hybrid rice LYPJ exposed to drought stress probed by chlorophyll a fluorescence transient. Plant Growth Regul. 81, 489–499. doi: 10.1007/s10725-016-0226-3

[B191] Wang F. Zhang X. Zhang S. Zhang S. Sun Y. (2020a). Interactions of microplastics and cadmium on plant growth and arbuscular mycorrhizal fungal communities in an agricultural soil. Chemosphere 254, 126791. doi: 10.1016/j.chemosphere.2020.126791 32320834

[B192] Weber A. Scherer C. Brennholt N. Reifferscheid G. Wagner M. (2018). PET microplastics do not negatively affect the survival, development, metabolism and feeding activity of the freshwater invertebrate Gammarus pulex. Environ. Pollut. 234, 181–189. doi: 10.1016/j.envpol.2017.11.014 29175683

[B193] Weithmann N. Möller J. N. Löder M. G. Piehl S. Laforsch C. Freitag R. (2018). Organic fertilizer as a vehicle for the entry of microplastic into the environment. Sci. Adv. 4 (4), eaap8060. doi: 10.1126/sciadv.aap8060 29632891 PMC5884690

[B194] Wong J. K. H. Lee K. K. Tang K. H. D. Yap P. S. (2020). Microplastics in the freshwater and terrestrial environments: Prevalence, fates, impacts and sustainable solutions. Sci. total Environ. 719, 137512. doi: 10.1016/j.scitotenv.2020.137512 32229011

[B195] Wu X. Hou H. Liu Y. Yin S. Bian S. Liang S.. (2022). Microplastics affect rice (Oryza sativa L.) quality by interfering metabolite accumulation and energy expenditure pathways: A field study. J. Hazardous Mater. 422, 126834. doi: 10.1016/j.jhazmat.2021.126834 34390954

[B196] Wu X. Liu Y. Yin S. Xiao K. Xiong Q. Bian S.. (2020). Metabolomics revealing the response of rice (Oryza sativa L.) exposed to polystyrene microplastics. Environ. Pollut. 266, 115159. doi: 10.1016/j.envpol.2020.115159 32663678

[B197] Wu J. Liu W. Zeb A. Lian J. Sun Y. Sun H. (2021). Polystyrene microplastic interaction with Oryza sativa: toxicity and metabolic mechanism. Environ. Sci.: Nano 8 (12), 3699–3710. doi: 10.1039/D1EN00636C

[B198] Xia T. Lin Y. Li S. Yan N. Xie Y. He M.. (2021). Co-transport of negatively charged nanoparticles in saturated porous media: Impacts of hydrophobicity and surface O-functional groups. J. Hazardous Mater. 409, 124477. doi: 10.1016/j.jhazmat.2020.124477 33172676

[B199] Xiao H. Lin Q. Li G. Zhao X. Li J. Li E. (2022). Comparison of biochar properties from 5 kinds of halophyte produced by slow pyrolysis at 500 °C. Biochar 4, 12. doi: 10.1007/s42773-022-00141-6

[B200] Xu B. Liu F. Cryder Z. Huang D. Lu Z. He Y.. (2020). Microplastics in the soil environment: Occurrence, risks, interactions and fate – A review. Crit. Rev. Environ. Sci. Technol. 50, 2175–2222. doi: 10.1080/10643389.2019.1694822

[B201] Xu Y. H. Liu R. Yan L. Liu Z. Q. Jiang S. C. Shen Y. Y.. (2012). Light-harvesting chlorophyll a/b-binding proteins are required for stomatal response to abscisic acid in Arabidopsis. J. Exp. Bot. 63 (3), 1095–1106. doi: 10.1093/jxb/err315 22143917 PMC3276081

[B202] Xu G. Liu Y. Yu Y. (2021). Effects of polystyrene microplastics on uptake and toxicity of phenanthrene in soybean. Sci. Total Environ. 783, 147016. 33872902 10.1016/j.scitotenv.2021.147016

[B203] Xu C. Wang H. Zhou L. Yan B. (2023). Phenotypic and transcriptomic shifts in roots and leaves of rice under the joint stress from microplastic and arsenic. J. Hazardous Mater. 447, 130770. doi: 10.1016/j.jhazmat.2023.130770 36640509

[B204] Xu Z. Zhang Y. Lin L. Wang L. Sun W. Liu C.. (2022). Toxic effects of microplastics in plants depend more by their surface functional groups than just accumulation contents. Sci. Total Environ. 833, 155097. doi: 10.1016/j.scitotenv.2022.155097 35421496

[B205] Yadav S. Gupta E. Patel A. Srivastava S. Mishra V. K. Singh P. C.. (2022). Unravelling the emerging threats of microplastics to agroecosystems. Rev. Environ. Sci. Biotechnol. 21, 771–798. doi: 10.1007/s11157-022-09621-4

[B206] Yang S.-S. Ding M.-Q. He L. Zhang C.-H. Li Q.-X. Xing D.-F.. (2021b). Biodegradation of polypropylene by yellow mealworms (*Tenebrio molitor*) and superworms (*Zophobas atratus*) via gut-microbe-dependent depolymerization. Sci. Total Environ. 756, 144087. doi: 10.1016/j.scitotenv.2020.144087 33280873

[B207] Yang C. Gao X. (2022). Impact of microplastics from polyethylene and biodegradable mulch films on rice (Oryza sativa L.). Sci. Total Environ. 828, 154579. doi: 10.1016/j.scitotenv.2022.154579 35302020

[B208] Yang J. Li R. Zhou Q. Li L. Li Y. Tu C.. (2021a). Abundance and morphology of microplastics in an agricultural soil following long-term repeated application of pig manure. Environ. Pollut. 272, 116028. doi: 10.1016/j.envpol.2020.116028 33199067

[B209] Yi Z. Zhang Z. Chen G. Rengel Z. Sun H. (2023). Microplastics have rice cultivar-dependent impacts on grain yield and quality, and nitrogenous gas losses from paddy, but not on soil properties. J. Hazardous Mater. 446, 130672. doi: 10.1016/j.jhazmat.2022.130672 36580778

[B210] Yi M. Zhou S. Zhang L. Ding S. (2021). The effects of three different microplastics on enzyme activities and microbial communities in soil. Water Environ. Res. 93, 24–32. doi: 10.1002/wer.1327 32187766

[B211] Yildiztugay E. Ozfidan-Konakci C. Arikan B. Alp F. N. Elbasan F. Zengin G.. (2022). The hormetic dose-risks of polymethyl methacrylate nanoplastics on chlorophyll a fluorescence transient, lipid composition and antioxidant system in Lactuca sativa. Environ. Pollut. 308, 119651. doi: 10.1016/j.envpol.2022.119651 35752396

[B212] Yu Y. Li J. Song Y. Zhang Z. Yu S. Xu M.. (2022). Stimulation versus inhibition: The effect of microplastics on pak choi growth. Appl. Soil Ecol. 177, 104505. doi: 10.1016/j.apsoil.2022.104505

[B213] Yu H. Zhang X. Hu J. Peng J. Qu J. (2020). Ecotoxicity of polystyrene microplastics to submerged carnivorous Utricularia vulgaris plants in freshwater ecosystems. Environ. Pollut. 265, 114830. doi: 10.1016/j.envpol.2020.114830 32540562

[B214] Yuan J. Ma J. Sun Y. Zhou T. Zhao Y. Yu F. (2020). Microbial degradation and other environmental aspects of microplastics/plastics. Sci. Total Environ. 715, 136968. doi: 10.1016/j.scitotenv.2020.136968 32014782

[B215] Zaid A. Mohammad F. Wani S. H. Siddique K. M. H. (2019). Salicylic acid enhances nickel stress tolerance by up-regulating antioxidant defense and glyoxalase systems in mustard plants. Ecotoxicol. Environ. Saf. 180, 575–587. doi: 10.1016/j.ecoenv.2019.05.042 31129436

[B216] Zeb A. Liu W. Meng L. Lian J. Wang Q. Lian Y.. (2022). Effects of polyester microfibers (PMFs) and cadmium on lettuce (Lactuca sativa) and the rhizospheric microbial communities: A study involving physio-biochemical properties and metabolomic profiles. J. Hazardous Mater. 424, 127405. doi: 10.1016/j.jhazmat.2021.127405 34629197

[B217] Zha Q. Xi X. He Y. Yin X. Jiang A. (2021). Effect of short-time high-temperature treatment on the photosynthetic performance of different heat-tolerant grapevine cultivars. Photochem. Photobiol. 97, 763–769. doi: 10.1111/php.13381 33458838

[B218] Zhang G. S. Liu Y. F. (2018). The distribution of microplastics in soil aggregate fractions in southwestern China. Sci. Total Environ. 642, 12–20. doi: 10.1016/j.scitotenv.2018.06.004 29894871

[B219] Zhang T. Luo X.-S. Xu J. Yao X. Fan J. Mao Y.. (2023). Dry–wet cycle changes the influence of microplastics (MPs) on the antioxidant activity of lettuce and the rhizospheric bacterial community. J. Soils Sediments 23, 2189–2201. doi: 10.1007/s11368-023-03479-x

[B220] Zhang Y. Zhang C. Jiang M. Zhou G. (2022). Bio-effects of bio-based and fossil-based microplastics: Case study with lettuce-soil system. Environ. Pollut. 306, 119395. doi: 10.1016/j.envpol.2022.119395 35525514

[B221] Zhang Q. Zhao M. Meng F. Xiao Y. Dai W. Luan Y. (2021). Effect of polystyrene microplastics on rice seed germination and antioxidant enzyme activity. Toxics 9, 179. doi: 10.3390/toxics9080179 34437497 PMC8402430

[B222] Zhao Y. Hu C. Wang X. Qing X. Wang P. Zhang Y.. (2019). Selenium alleviated chromium stress in Chinese cabbage (Brassica campestris L. ssp. Pekinensis) by regulating root morphology and metal element uptake. Ecotoxicol. Environ. Saf. 173, 314–321. doi: 10.1016/j.ecoenv.2019.01.090 30784794

[B223] Zhou C.-Q. Lu C.-H. Mai L. Bao L.-J. Liu L.-Y. Zeng E. Y. (2021). Response of rice (Oryza sativa L.) roots to nanoplastic treatment at seedling stage. J. Hazardous Mater. 401, 123412. doi: 10.1016/j.jhazmat.2020.123412 32763702

[B224] Zhou W. Wang Q. Wei Z. Jiang J. Deng J. (2023). Effects of microplastic type on growth and physiology of soil crops: Implications for farmland yield and food quality. Environ. Pollut. 326, 121512. doi: 10.1016/j.envpol.2023.121512 36967010

[B225] Ziajahromi S. Kumar A. Neale P. A. Leusch F. D. L. (2018). Environmentally relevant concentrations of polyethylene microplastics negatively impact the survival, growth and emergence of sediment-dwelling invertebrates. Environ. Pollut. 236, 425–431. doi: 10.1016/j.envpol.2018.01.094 29414367

